# Suppressing Polysulfide Shuttle and Boosting Reaction Kinetics via UiO‐66‐SH‐Functionalized Separators for Lithium–Sulfur Batteries

**DOI:** 10.1002/smsc.70382

**Published:** 2026-08-21

**Authors:** Min Chen, Yaping Lu, Xinmin Zhang, Yixin Song, Fengtao Shao, Xuetao Gao, Zhichao Liu, Chunmei Xu, Yiting Peng, Yaping Feng, Kai Xi, Yatao Liu

**Affiliations:** ^1^ Beijing Advanced Innovation Center for Soft Matter Science and Engineering Beijing University of Chemical Technology Beijing China; ^2^ College of Materials Science and Engineering Beijing University of Chemical Technology Beijing China; ^3^ State Key Laboratory of Organic‐Inorganic Composites College of Chemical Engineering Beijing University of Chemical Technology Beijing China; ^4^ Shanghai Key Laboratory of Materials Protection and Advanced Materials in Electric Power Shanghai University of Electric Power Shanghai China; ^5^ School of Chemistry State Key Laboratory of Electrical Insulation and Power Equipment Xi’an Jiaotong University Xi’an China

**Keywords:** Li–S batteries, lithium anode, polysulfides shuttle, sulfur reaction kinetics, UiO‐66‐SH functionalized separator

## Abstract

Lithium–sulfur (Li–S) batteries represent promising energy storage devices by virtue of their ultrahigh theoretical energy density, yet their practical deployment is severely impeded by the polysulfide shuttle, sluggish sulfur redox kinetics, and progressive degradation of lithium anodes. Herein, we design a thiol‐functionalized UiO‐66 (UiO‐66‐SH)‐coated polypropylene separator that addresses these challenges simultaneously. The UiO‐66‐SH combines abundant coordinatively unsaturated Zr sites and —SH groups, enabling both strong chemical anchoring of lithium polysulfides and catalytic acceleration of their conversion to Li_2_S. The modified separator exhibits superior electrolyte wettability, a high Li^+^ transference number (0.76), and enhanced ionic conductivity. Furthermore, it regulates lithium deposition to achieve a dendrite‐free anode and mitigates polysulfide‐induced corrosion. Consequently, Li–S cells incorporating UiO‐66‐SH/PP separator deliver an initial discharge capacity of 821.3 mAh g^−1^ at 1 C, retain 521 mAh g^−1^ after 500 cycles, and achieve a high‐rate capacity of 590.9 mAh g^−1^ at 5 C. Even under a high sulfur loading of 5.05 mg cm^−2^, a reversible capacity of 620.1 mAh g^−1^ is maintained after 200 cycles. This work offers a rational design of a multifunctional MOF‐based separator that integrates polysulfide entrapment, catalytic conversion, and anode protection, paving the way toward high‐performance, long‐life Li–S batteries.

## Graphical abstract

This work demonstrates a UiO‐66‐SH‐functionalized separator that boosts Li^+^ transport, thermal stability, polysulfide anchoring, and sulfur redox kinetics in Li–S batteries. As a result, the cell delivers an initial capacity of 821.3 mAh g^−1^ at 1 C and retains 521 mAh g^−1^ after 500 cycles. Even at a high sulfur loading of 5.05 mg cm^−2^, a reversible capacity of 620.1 mAh g^−1^ is achieved after 200 cycles.

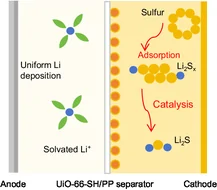

## 1 Introduction

With the rapid advancement of science and technology, coupled with growing concerns over energy scarcity and environmental degradation [1, 2], there is an urgent need for high‐energy‐density storage systems in electric vehicles and portable electronics. Conventional lithium‐ion batteries (LIBs) are approaching their theoretical energy density limit (300–400 Wh kg^−1^), rendering them increasingly inadequate for the evolving energy storage market. Thus, developing next‐generation battery technologies with higher energy density has become a critical research priority [3, 4, 5]. Among various candidates, lithium–sulfur (Li–S) batteries have attracted extensive attention owing to their high theoretical energy density (up to 2600 Wh kg^−1^), abundant sulfur resources, and environmental friendliness [6, 7, 8, 9].

However, several key challenges impede the practical implementation of Li–S batteries. First, both elemental sulfur and its final discharge product (Li_2_S) are intrinsically poor conductors [10, 11]. This severely hinders electron and ion transport, resulting in low sulfur utilization and sluggish redox kinetics [12, 13]. Second, during discharge, the cleavage of S—S bonds produces soluble lithium polysulfides (LiPSs) in typical ether‐based electrolytes, giving rise to the notorious “shuttle effect” [14, 15, 16]. These dissolved LiPSs migrate to the lithium metal anode, where they undergo irreversible parasitic reactions, causing active material loss, rapid capacity fading, reduced Coulombic efficiency, and severe corrosion of the lithium anode [15, 16, 17, 18]. Collectively, these issues hamper the commercialization of Li–S batteries. Moreover, conventional polyolefin‐based separators (e.g., polypropylene and polyethylene) suffer from inherent drawbacks such as limited mechanical strength and poor thermal stability [3, 19].

To address these challenges, numerous strategies have been explored, including porous carbon materials for physical confinement of LiPSs, polar materials for chemical adsorption, electrocatalysts to promote the conversion of LiPSs to Li_2_S, and separator modifications to block dissolved LiPSs [20, 21, 22, 23, 24]. Early work often employed porous carbons to physically encapsulate LiPSs via size‐exclusion effects. However, due to weak interactions and limited confinement, non‐polar carbons can only partially restrict LiPS diffusion [25]. Polar materials such as metal compounds, polymers, and heteroatom‐doped carbons anchor LiPSs through stronger chemical interactions, partially mitigating their dissolution and diffusion [26, 27, 28]. Nevertheless, their finite adsorption sites and inability to accelerate conversion kinetics often result in unsatisfactory performance. Separator modification, which integrates functional materials onto separators by coating or vacuum filtration, offers a straightforward, efficient, and scalable approach. The introduced functional materials can regulate ion transport, facilitating Li^+^ migration while suppressing the shuttle effect [29, 30].

Recently, research focus has shifted from merely “suppressing LiPS shuttling” to “accelerating the conversion kinetics between LiPSs and Li_2_S through catalysis”, which is regarded as a fundamental solution to the shuttle issue and a key to achieving long cycle life [31, 32]. Moderate interactions between catalysts and LiPSs have been shown to effectively alleviate capacity decay by catalyzing the transformation of LiPSs to Li_2_S and suppressing the shuttle effect [33, 34, 35]. Metal–organic frameworks (MOFs), sulfides, and defect‐engineered materials have been employed to promote the liquid‐to‐solid conversion of LiPSs [36, 37, 38]. MOF‐based catalysts, in particular, have drawn considerable interest due to their ultrahigh specific surface area, abundant metal active sites, tunable pore sizes, and excellent chemical/thermal stability [39, 40, 41]. For instance, Cai et al. reported a hybrid catalyst consisting of cerium‐based MOFs and carbon nanotubes (CNTs), in which the conductive CNTs and MOFs with rich metal sites act synergistically to achieve efficient LiPSs anchoring and catalytic conversion [42]. Although such strategies can suppress the shuttle effect and accelerate Li_2_S conversion, they often overlook the functional trade‐offs inherent in single‐component systems. An ideal catalyst should balance adsorption, catalytic conversion, and desorption capabilities [43]. Previous studies have revealed a volcano‐type relationship between LiPSs adsorption strength and catalytic activity; materials with strong catalytic activity typically exhibit weak adsorption capacity [44]. Therefore, the rational design of Li–S catalysts must consider the interplay between adsorption and catalysis. Despite these advances, conventional MOF‐modified separators still face critical limitations. Single‐component coatings seldom integrate strong polysulfide adsorption, efficient catalytic conversion, and lithium anode protection into one system, leaving the shuttle effect and dendrite growth only partially suppressed. Thus, developing a multifunctional separator that simultaneously addresses these intertwined challenges remains highly desirable.

The advantages of MOF materials as separator modifiers can be summarized in three aspects [45]: (1) the unique porous structure of MOFs serves as a physical barrier to suppress the shuttling of lithium polysulfides, and reducing the loss of active sulfur species; (2) the ordered pore channels of MOFs enhance ion transport, and their high porosity enables electrolyte storage, which lowers interfacial impedance and improves ionic conductivity; (3) the abundant active sites in MOFs provide chemical adsorption and catalytic effects toward lithium polysulfides. The strong affinity between the Lewis acid metal centers of MOFs and the Lewis basic polysulfide species gives rise to chemisorption (coordination) or catalytic effects. Furthermore, strategies such as ligand defect engineering, metal doping, and targeted functional group introduction can expose more active sites, effectively enhancing the chemical interaction and catalytic activity of MOFs toward polysulfides [46, 47]. Existing MOF‐based separator functionalization strategies mostly focus on enhancing a single function. For example, UiO‐66‐NH_2_ prioritizes ion transport and adsorption [48], UiO‐66‐F_4_ emphasizes adsorption [49], and UiO‐66(SO_3_Li)_4_ focuses on electrostatic repulsion and Li^+^ conduction [50]. Few reported materials can simultaneously achieve polysulfide adsorption, catalytic conversion, and lithium anode protection [51, 52].

**FIGURE 1:**
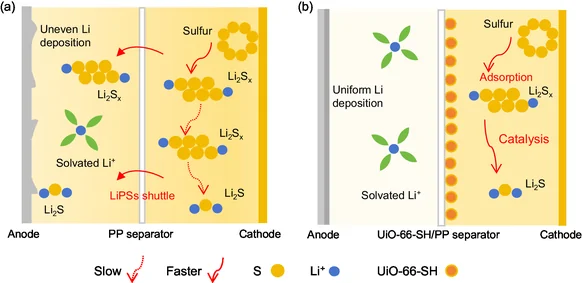
Schematic illustration of the mechanism of UiO‐66‐SH functionalized PP separator. (a) Li–S cells using traditional PP separators exhibit severe polysulfides shuttle, sluggish sulfur conversion kinetics and lithium corrosion/dendrites. (b) UiO‐66‐SH functionalized PP separator can effectively restrict the lithium shuttle, enhance the sulfur reaction kinetics, and enable uniform Li deposition without corrosion/dendrites.

Herein, we report a thiol‐functionalized UiO‐66 (UiO‐66‐SH) modified polypropylene separator that simultaneously tackles the polysulfide shuttle, sluggish sulfur redox kinetics, and lithium anode instability (Figure 1). The design rationale is twofold: (i) the coordinatively unsaturated Zr sites in UiO‐66 provide strong Lewis acid centers for chemical anchoring of lithium polysulfides, and (ii) the free —SH groups further enhance polysulfide adsorption and participate in the catalytic conversion of soluble LiPSs to insoluble Li_2_S. Moreover, the ordered microporous architecture of UiO‐66‐SH homogenizes Li^+^ flux, suppresses dendritic Li deposition, and improves the thermal and mechanical robustness of the separator. As a result, Li–S cells using the UiO‐66‐SH/PP separator deliver an outstanding initial discharge capacity of 821.3 mAh g^−1^ at 1 C and retain 521 mAh g^−1^ after 500 cycles, with an average capacity decay of only 0.066% per cycle. The cell also exhibits superior rate capability, achieving 590.9 mAh g^−1^ at 5 C. Even under a high sulfur loading of 5.05 mg cm^−2^, a reversible capacity of 620.1 mAh g^−1^ is retained after 200 cycles at 0.2 C. Furthermore, Li||Li symmetric cells with the modified separator operate stably for over 600 h without dendrite formation, and post‐mortem analysis reveals drastically reduced sulfur residue (2.27 at%) on the lithium anode, confirming effective polysulfide interception. This work demonstrates a rational strategy to transform a commercial separator into a multifunctional platform that integrates polysulfide entrapment, catalytic conversion, and anode protection, offering a promising pathway toward practical high‐energy Li–S batteries.

## 2 Results and Discussion

### 2.1 Characterization of UiO‐66‐SH/PP Separators

**FIGURE 2:**
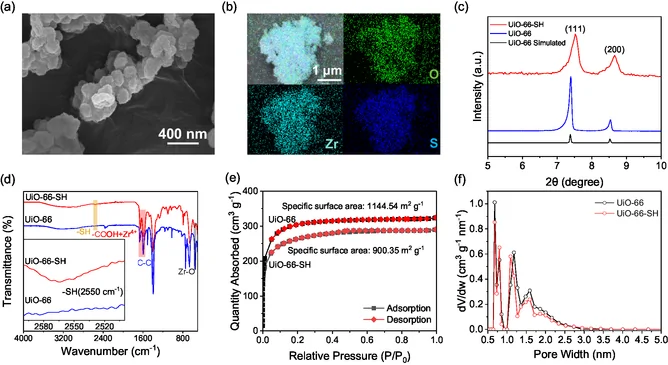
Structural characterization of the UiO‐66‐SH. (a) Scanning electron microscopy (SEM) images of UiO‐66‐SH. (b) EDS elemental distribution mapping of UiO‐66‐SH. (c) XRD patterns focusing on (111) and (200) peak and (d) FTIR spectra of UiO‐66 and UiO‐66‐SH. (e) N_2_ sorption isotherm of UiO‐66 and UiO‐66‐SH. (f) The pore size distribution of UiO‐66 and UiO‐66‐SH.

The thiol‐modified UiO‐66 (UiO‐66‐SH) was synthesized via hydrothermal reaction of zirconium chloride (ZrCl_4_) and 2, 4‐mercaptobenzenedicarboxylic acid (H_2_BDC‐SH). After synthesis, UiO‐66‐SH was activated at 150 °C under vacuum to generate coordinatively unsaturated Zr sites, and then dispersed into a slurry and coated onto a commercial polypropylene (PP) separator (Figure S1) [53]. UiO‐66‐SH exhibits a nanoparticle morphology (Figure 2a), with uniform dispersion and no significant agglomeration, aligning with typical morphological features of UiO‐66‐type MOFs [54]. Energy‐dispersive X‐ray spectroscopy (EDS) mapping (Figure 2b) reveals homogeneous distribution of Zr, O, and S elements. This uniformity ensures consistent active sites across the material surface, preventing local variations in polysulfide adsorption/catalytic performance and thereby improving the overall functionality of the modified separator [54, 55]. The crystal structure of UiO‐66‐SH was characterized by X‐ray diffraction (XRD). As shown in Figures 2c and S2, XRD analysis reveals that both materials retain the same fcu topology and characteristic diffraction peaks [56], confirming that the incorporation of —SH does not disrupt the crystal structure of UiO‐66; UiO‐66‐SH exhibits only a slight low‐angle shift of approximately 0.1°–0.2° due to the steric hindrance of the —SH groups, indicating a very small spacing layer difference. In addition, the crystallite sizes associated with the (111) and (002) diffraction reflections of the MOF samples were determined using the Scherrer equation [45, 57]. For UiO‐66‐SH, the crystallite sizes derived from the (111) and (002) planes are 24.5 and 21.1 nm, respectively, substantially smaller than those of pristine UiO‐66 (71.6 and 72.5 nm, respectively). This reduction in crystallite size suggests that the incorporation of —SH groups possibly introduces additional structural defects into the MOF framework, which may provide more active sites for lithium polysulfide adsorption and catalytic conversion. The XRD pattern of the UiO‐66‐SH/PP composite separator displays characteristic peaks of both UiO‐66‐SH and PP (Figure S2), confirming the successful coating of UiO‐66‐SH onto the PP separator without compromising its crystalline structure [58].

Fourier transform infrared (FTIR) spectroscopy (Figure 2d) verifies the functional groups in UiO‐66‐SH. The peak near 2550 cm^−1^ corresponds to the stretching vibration of the thiol group (—SH), the band at 1500 cm^−1^ is assigned to C—C skeletal vibrations of the benzene ring in the organic linker, and the signal around 1700 cm^−1^ arises from the coordination between carboxylate groups (—COOH) and Zr^4+^ ions [59, 60]. The pore structure of UiO‐66‐SH was evaluated using N_2_ adsorption desorption isotherms. The isotherm exhibits a Type I profile, indicative of a microporous structure, which was further confirmed by the pore size distribution shown in Figure 2e,f. The Brunauer–Emmett–Teller (BET) specific surface area of UiO‐66‐SH was determined to be ~900.3 m^2^ g^−1^, lower than that of pristine UiO‐66 (1144.5 m^2^ g^−1^). Despite this moderate reduction, UiO‐66‐SH still retains a considerably high surface area, which is essential for maintaining favorable electrolyte wettability and adequate porosity of the composite separator. The decrease in surface area can be attributed to the introduction of thiol groups, which reduces the crystallinity of the MOF (as evidenced by XRD analysis) and consequently leads to a less ordered pore structure. This structural evolution indirectly indicates an increase in defect density within the UiO‐66‐SH framework. Importantly, the presence of such defects is highly beneficial, as they provide abundant active sites for both the adsorption and catalytic conversion of lithium polysulfides. This defect‐mediated functionality is a key factor underlying the superior electrochemical performance of UiO‐66‐SH over pristine UiO‐66, particularly in enhancing the cycling stability of lithium–sulfur batteries [61].

As depicted in Figure S3, TGA profiles confirm that both MOF materials exhibit robust thermal stability at elevated temperatures. Compared with pristine UiO‐66, the incorporation of thiol (–SH) moieties into the framework linkers increases the density of coordinatively unsaturated sites on the Zr_6_‐based secondary building units. These undercoordinated Zr centers act as preferential initiation sites for thermal decomposition, accelerating framework collapse at high temperatures and consequently leading to a moderate decline in the overall thermal stability of UiO‐66‐SH. Despite this modest reduction, the decomposition temperature of UiO‐66‐SH still far exceeds that of conventional polyolefin separators, which is critical for ensuring the thermal safety of the composite separator. Moreover, this slight decrease in thermal stability is consistent with the XRD results, which reveal reduced crystallite sizes and increased defect densities upon thiol functionalization, further supporting the structural origin of the observed thermal behavior. Coordinatively unsaturated Zr sites are well established to play a critical role in the chemical adsorption and coordination of LiPSs. XPS was employed to characterize the chemical states of the two MOF materials. As shown in Figure S4, the Zr 3d peaks of UiO‐66‐SH shift to higher binding energies relative to those of pristine UiO‐66. This positive shift indicates a decrease in the electron density around the Zr centers, which can be attributed to the generation of additional coordinatively unsaturated Zr sites upon thiol functionalization. The creation of these defect sites, likely arising from partial ligand exchange or structural distortion during –SH incorporation, is expected to enhance the Lewis acidity of the Zr clusters and provide more active sites for LiPSs anchoring and conversion [62]. Furthermore, static lithium polysulfide adsorption tests on the MOF powders corroborate the distinct difference in LiPS trapping performance arising from –SH functionalization. As visualized in Figure S5, after an identical adsorption period, the LiPS solution containing dispersed UiO‐66‐SH powder becomes clear and transparent, whereas the solution with UiO‐66 powder retains a deep yellow color.

**FIGURE 3:**
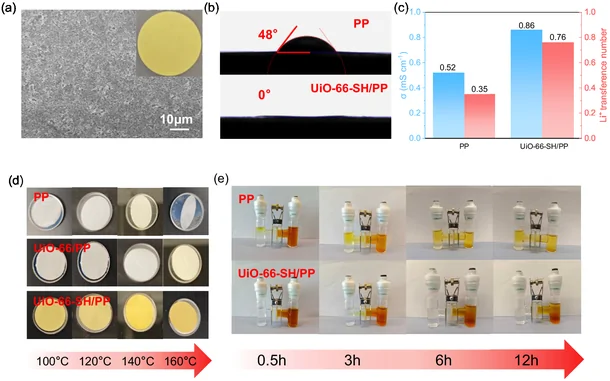
Properties of the UiO‐66‐SH/PP composite separator. (a) SEM image of surface morphology of UiO‐66‐SH/PP composite separator. (b) Electrolyte wetting tests showing the contact angle of electrolyte on PP and UiO‐66‐SH/PP separators. (c) Ionic conductivity and Li^+^ transference number. Note that this ionic conductivity value represents the collective conductivity of the separator and the electrolyte, not the intrinsic conductivity of the electrolyte alone. (d) Photos of thermal shrinkage tests for different separators. (e) Photographs of polysulfide permeation measurements for PP and UiO‐66‐SH/PP separators.

Figure 3a shows the surface morphology of the UiO‐66‐SH/PP composite separator. The surface view reveals a uniform distribution of UiO‐66‐SH particles, forming a smooth and continuous coating with a thickness of approximately 15 μm (Figure S6). Effective electrolyte‐separator contact is crucial for rapid Li^+^ transport in Li–S batteries. Contact angle measurements (Figure 3b) demonstrate improved wetting behavior for the modified separator. The UiO‐66‐SH‐coated separator absorbs the electrolyte almost instantaneously upon droplet deposition, superior to the UiO‐66/PP separator (~10°) (Figure S7) and the bare PP separator (~48°) (Figure 3b). A separator with excellent electrolyte affinity can reduce ionic transfer resistance and increase the rate capacity of lithium–sulfur batteries. As shown in Figure S8, UiO‐66/PP (81.5% and 234.1%) and UiO‐66‐SH/PP (78.2% and 205.8%) separators have higher porosity and electrolyte uptake than the PP separator (54.7% and 146.5%). This performance improvement originates from the intrinsically high specific surface area, inherent porous structure, and favorable electrolyte affinity of the MOF materials. Thus, the electrolyte uptake of the UiO‐66/PP and UiO‐66‐SH/PP composite membranes is more suitable for separators in battery compared to the PP membranes.

Further, the cell assembled with the UiO‐66‐SH coating enables higher ionic conductivity (0.86 mS cm^−1^) and lithium‐ion transference number (0.76) (Figures 3c, S9 and S10), indicating enhanced Li^+^ transport behaviors. The enhanced ion transport kinetics of UiO‐66‐SH arise from three key factors: (1) unsaturated metal sites immobilize TFSI anions, increasing the Li^+^ transference number; (2) high porosity and a polar surface improve electrolyte wettability and retention, forming continuous ion‐conducting pathways that enhance ionic conductivity; (3) ordered nanopores serve as low‐resistance channels for Li^+^ diffusion while weakening Li^+^–solvent and Li^+^–anion interactions, thereby facilitating desolvation and promoting selective Li^+^ transport.

As separators are key components affecting battery thermal safety, we evaluated the thermal stability of the UiO‐66‐SH/PP composite separator. Compared with the commercial PP separator, the UiO‐66‐SH/PP composite separator shows notably enhanced thermal resistance. Differential scanning calorimetry (DSC) curves reveal that the PP separator exhibits an endothermic peak starting at 140.23 °C, corresponding to the melting and pore closure of polypropylene, whereas the UiO‐66‐SH/PP separator initiates thermal melting at 151.67 °C (Figure S11). The endothermic enthalpy change of the PP separator is 124.06 J g^−1^, whereas that of the UiO‐66‐SH/PP separator is 113.25 J g^−1^. These results indicate that the MOF coating effectively increases the melting temperature of the separator, demonstrating enhanced thermal stability of the MOF. Thermal shrinkage tests (Figure 3d) further confirm this improvement: after sequential heating at 100, 120, 140, and 160 °C for 1 h, the PP separator shows obvious shrinkage at 140 °C, while the composite separators remain dimensionally stable, with the UiO‐66‐SH variant displaying particularly good flame‐retardant characteristics. Furthermore, compared with the PP separator, the UiO‐66‐SH modified separator exhibits a higher fracture stress, demonstrating superior mechanical properties. (Figure S12).

We further performed a polysulfide permeation test to evaluate the ability of different separators to block lithium polysulfides (Figures 3e and S13). For the pristine PP separator, Li_2_S_6_ readily diffused through the membrane within 0.5 h, turning the opposite side of the device yellow, whereas the UiO‐66‐modified separator effectively suppressed polysulfide permeation for 3 h. In contrast, the UiO‐66‐SH‐modified separator caused negligible color change, confirming its effective adsorption of lithium polysulfides and remarkable suppression of the shuttle effect. Static adsorption tests were also conducted by immersing PP, UiO‐66/PP, and UiO‐66‐SH/PP separators in 1 mM Li_2_S_6_ solution for 3 days (Figure S14). Visual inspection showed that the solution containing UiO‐66‐SH/PP became nearly transparent, whereas the solution with pristine PP remained bright yellow, highlighting the weak affinity of bare PP for lithium polysulfides. UV–vis spectroscopy further confirmed this difference: the characteristic absorption peak of S_4_^2−^ at 410 nm completely disappeared in the UiO‐66‐SH/PP group, indicating the strongest polysulfide adsorption capacity, which facilitates the subsequent catalytic conversion of soluble lithium polysulfides.

Shuttle current measurements were performed on Li–S cells using different separators at a constant charging voltage of 2.4 V, the characteristic potential for shuttle current generation in practical Li–S batteries [63]. The shuttle current of UiO‐66‐modified separator decreased significantly (3.98 μA cm^−2^), and the UiO‐66‐SH‐modified separator exhibited the lowest shuttle current of 2.85 μA cm^−2^, far lower than that of the pristine PP counterpart (13.05 μA cm^−2^)—only one‐fifth of the latter value (Figure S15). This result further highlights the superior polysulfide anchoring ability of UiO‐66‐SH, which effectively suppresses the polysulfide shuttle.

**FIGURE 4:**
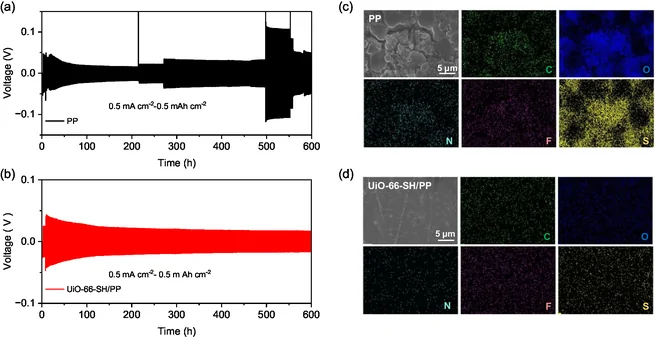
The effects of UiO‐66‐SH/PP composite separator on lithium anode. (a,b) Cycling performance of Li|Li symmetric cells: (a) PP separator and (b) UiO‐66‐SH/PP composite separator. (c,d) EDS elemental distribution map of lithium anode from lithium–sulfur cells cycled at 0.5 C for 100 cycles: (c) PP separator and (d) UiO‐66‐SH/PP composite separator.

To evaluate the anode interfacial stability enabled by the UiO‐66‐SH modified separator, lithium symmetric cells were cycled at a current density of 0.5 mA cm^−2^. As displayed in Figures 4a,b and S16, the cell assembled with the UiO‐66‐SH composite separator delivers a stable voltage profile with low overpotential within 600 h, while the UiO‐66 composite separator maintains stable cycling for 400 h. In contrast, the Li|Li cells with pristine PP separator presents severe voltage polarization. This indicates that UiO‐66‐SH coating significantly enhances the lithium deposition behavior. Li–Cu half‐cells were further fabricated to investigate the lithium deposition morphology. After deposition at either a current density of 0.5 mA cm^−2^ with an areal capacity of 4 mAh cm^−2^ or a current density of 1 mA cm^−2^ with an areal capacity of 4 mAh cm^−2^, more pronounced lithium dendrites are observed in cells with a bare PP separator (Figure S17a–d). By comparison, the cells with UiO‐66‐SH functionalized separator achieve uniform and dendrite‐free Li deposition. Benefiting from the inherent 3D porous structure of UiO‐66‐SH, the Li^+^ flux is effectively homogenized to induce homogeneous Li plating. This demonstrates that the functionalized separator optimizes Li deposition uniformity and interfacial stability, thereby restraining dendrite proliferation and guaranteeing the long‐cycle stability and operational safety of Li–S batteries.

We further disassembled the cycled cells (100 cycles at 0.5 C) for post‐mortem analysis to further explore the inhibition effect of the UiO‐66‐SH/PP composite separator on the polysulfide shuttle and lithium anode degradation. The cycled Li anodes were subsequently characterized via SEM and EDS (Figure 4c,d). EDS results show that the sulfur content on the Li anode surface with the UiO‐66‐SH/PP separator is only ~2.27% (Tables S1 and S2), which is remarkably lower than ~8.76% for the pristine PP counterpart. This verifies the excellent lithium polysulfide trapping capability of UiO‐66‐SH. Moreover, the Li anode paired with UiO‐66‐SH exhibits a flat and intact surface without conspicuous dendrite growth, whereas the PP separator corresponds to rough surfaces owing to the corrosion of polysulfides. These results confirm that UiO‐66‐SH can effectively regulate Li deposition behavior and stabilize the solid electrolyte interphase of lithium metal anodes.

### 2.2 Redox Kinetics of Polysulfides on UiO‐66‐SH/PP Separators

**FIGURE 5:**
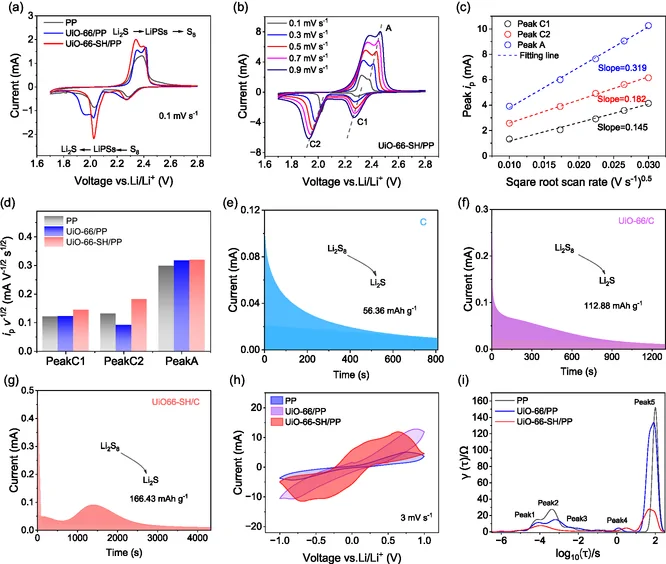
Evaluation of the sulfur reaction kinetics. (a) CV curves at a scanning rate of 0.1 mV s^−1^. (b) CV curves of UiO‐66‐SH/PP at a scanning rate of 0.1, 0.3, 0.5, 0.7, 0.9 mV s^−1^. (c) The peak current versus the square root of scanning rate of peaks A, C1, and C2 in different Li–S cells. (d) Slope of *i*_p_/*v* ^1/2^, where *i*_p_ and *v* refer to the peak current and scan rate of the CV curves, respectively. (e–g) The deposition of Li_2_S, time‐current curves of different catalysts under constant voltage discharge of 2.05 V. (h) CV curves of Li_2_S_6_ symmetric cells at 3 mV s^−1^. (i) EIS test analysis using distribution of relaxation times.

We next performed cyclic voltammetry (CV) at different scan rates to investigate the effects of UiO‐66‐SH coating on sulfur redox behavior (Figure 5a). The CV curves of all three cells exhibit two typical reduction peaks and two oxidation peaks. These two reduction peaks correspond to the solid–liquid–solid reduction process from solid S_8_ to soluble lithium polysulfides and finally to solid Li_2_S, while the two oxidation peaks represent the reverse conversion process [64, 65]. Notably, the cell with the UiO‐66‐SH‐coated separator shows sharper peaks with higher currents as compared with the cells employing PP separator and the UiO‐66/PP separator, revealing faster reaction kinetics of sulfur species in the presence of UiO‐66‐SH coating. The Li^+^ diffusion kinetics were then further examined by CV at various scan rates (Figures 5b–d, S18 and S19). Compared with the PP and UiO‐66/PP separator‐based cells, the Li–S cells with UiO‐66‐SH/PP separator exhibits steeper slopes for the linear fits of peak currents versus the square root of the scan rate at oxidation peak A and reduction peaks C1 and C2, implying faster Li^+^ diffusion, demonstrating that the UiO‐66‐SH effectively enhances ion‐transport kinetics during the discharge and charge of Li–S batteries [66].

As the reduction from soluble LiPSs to solid Li_2_S is rather sluggish, we then conducted Li_2_S deposition experiments to explore the effects of UiO‐66‐SH on Li_2_S conversion, using carbon‐coated aluminum (C‐Al) foil, UiO‐66‐coated Al foil, and UiO‐66‐SH‐coated Al foil (Figure 5e–g). The results show that UiO‐66‐SH significantly enhances the Li_2_S deposition capacity (166.43 mAh g^−1^) compared to the counterpart. Specifically, the higher Li_2_S deposition capacity of UiO‐66‐SH than UiO‐66 implies that the —SH group is efficient in enhancing the conversion process from soluble LiPSs to Li_2_S. Moreover, the reversibility of Li_2_S deposition‐dissolution was also evaluated (Figure S20). The results show that the C‐Al foil exhibits the weakest Li_2_S dissolution ability, while the UiO‐66‐SH displays the highest dissolution capability. The enhanced sulfur reaction kinetics were further evidenced by the Li_2_S_6_ symmetric cells (Figure 5h). At a scan rate of 3 mV s^−1^, the cell with the UiO‐66‐SH separator presents the highest current, in comparison with the cells with PP separator and UiO‐66/PP separator. Additional CV measurements at different scan rates (Figure S21a–d) reveal that the UiO‐66‐SH enables the highest current across all rates. These results demonstrate that UiO‐66‐SH is efficient in accelerating the LiPSs conversion reactions [67, 68].

We further utilized electrochemical impedance spectroscopy (EIS) to investigate the charge–transfer and ion‐diffusion kinetics. The Nyquist plots consist of a semicircle in the high‐frequency region (related to charge–transfer resistance) and a sloping line in the low‐frequency region (related to Li^+^ diffusion). The cell with the UiO‐66‐SH separator shows the smallest charge–transfer resistance (26.72 Ω, Figure S22), reflecting rapid Li^+^ transport in the presence of the anchoring‐catalyst separator [69]. The reaction processes of fresh cells were analyzed using distribution of relaxation times (DRT) derived from the EIS data (Figure 5i). The DRT spectra can be divided into three main regions: peak 1 (inter‐particle resistance), peaks 2–4 (cathode charge–transfer processes), and peak 5 (diffusion). Compared with the PP and UiO‐66 separators, the cell with the UiO‐66‐SH separator exhibits significantly lower peak intensities, especially for peak 5, indicating faster charge–transfer kinetics and reduced Li^+^ diffusion impedance. These results demonstrate that the UiO‐66‐SH catalyst effectively promotes the reaction kinetics of sulfur species.

### 2.3 Electrochemical Performance of UiO‐66‐SH/PP Separators

**FIGURE 6:**
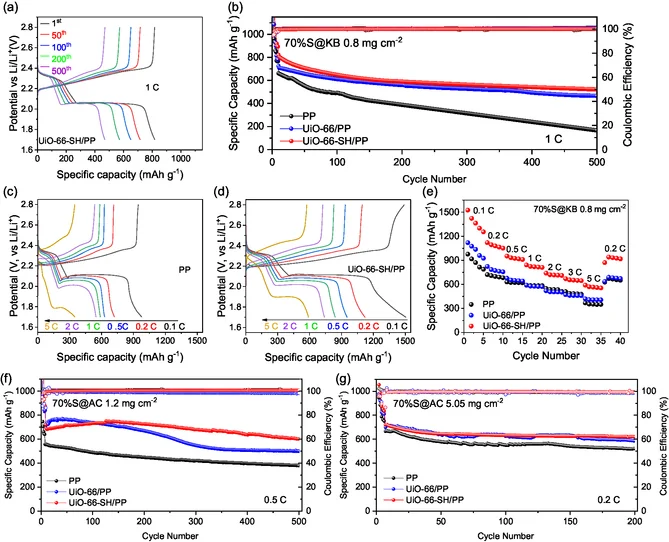
Evaluation of the rate capability and cycling stability of Li–S batteries with different separators. (a) Charge–discharge curves of UiO‐66‐SH/PP separator at 1 C under different cycle numbers. (b) Cycling stability of Li–S batteries with different separators at 1 C. (c) Charge discharge curves of PP separator at different rates. (d) Charge discharge curves of UiO‐66‐SH/PP separator at different rates. (e) Rate performance test from 0.1 to 5 C. (f) Cycling stability of Li–S batteries with different separators at 0.5 C. (g) Cycling stability of high S loading Li–S batteries with different separators at 0.2 C.

The electrochemical performance of Li–S cells assembled with pristine PP, UiO‐66/PP, and UiO‐66‐SH/PP separators was systematically evaluated to confirm the superiority of the MOF‐modified separators. Figure 6a shows the galvanostatic charge–discharge profiles of the UiO‐66‐SH/PP cell at the 1st, 50th, 100th, 200th, and 500th cycles. The cell maintains well‐defined and stable redox discharge plateaus throughout long‐term cycling, with relatively low voltage polarization and a moderate capacity decay rate. As presented in Figure 6b, the UiO‐66‐SH/PP cell delivers a high initial discharge capacity of 821.3 mAh g^−1^ at 1 C, and still retains a reversible capacity of 521 mAh g^−1^ after 500 cycles. In sharp contrast, cells with pristine PP and UiO‐66/PP separators retain only 166.04 and 470.99 mAh g^−1^, respectively, under identical conditions. This remarkable electrochemical superiority stems from the strong polysulfide adsorption and anchoring effect of UiO‐66‐SH, which effectively suppresses the shuttle effect, mitigates the loss of active sulfur species, and stabilizes the electrode interfacial structure – thereby greatly enhancing the long‐term cycling durability and capacity retention of Li–S batteries.

Benefiting from the effective adsorption and enhanced conversion kinetics of lithium polysulfides by the UiO‐66‐SH coating, the cell employing the UiO‐66‐SH/PP separator also exhibits outstanding rate capability. A comparison of the charge–discharge curves at various rates (Figures 6c,d and S23) reveals that the UiO‐66‐SH/PP cell maintains well‐defined discharge plateaus with minimal polarization, whereas cells with pristine PP and unmodified UiO‐66 separators suffer from severe polarization under the same conditions. Notably, the UiO‐66‐SH/PP cell achieves a specific capacity of 590.86 mAh g^−1^ at a high rate of 5 C, far exceeding the capacities of the PP (369.48 mAh g^−1^) and UiO‐66 (420.79 mAh g^−1^) cells (Figure 6e). This significant improvement underscores the critical role of the thiol‐functionalized UiO‐66‐SH in mitigating active material loss and facilitating rapid LiPSs redox within the cell [70].

Long‐term cycling performance at 0.5 C further demonstrates the superior stability of the UiO‐66‐SH cell (Figure 6f). After 500 cycles, it retains a reversible capacity of 600.92 mAh g^−1^, corresponding to a high capacity retention of 88.02% and an average capacity decay of only 0.024% per cycle. In contrast, cells with the PP and UiO‐66 separators deliver only 279.06 and 500.38 mAh g^−1^, with capacity retentions of 68.11% and 69.10%, respectively. These results highlight the pronounced advantage of the UiO‐66‐SH in suppressing the shuttle effect and stabilizing the sulfur cathode over prolonged cycling. High sulfur loading is essential for achieving practical energy density in Li–S batteries. With a sulfur loading of 5.05 mg cm^−2^, the UiO‐66‐SH cell delivers an initial discharge capacity of 719.61 mAh g^−1^ at 0.2 C (Figure 6g) and still retains 620.11 mAh g^−1^ after 200 cycles, significantly outperforming both the PP (495.56 mAh g^−1^) and the unmodified UiO‐66 (555.5 mAh g^−1^) cells. These findings further confirm the great potential of the UiO‐66‐SH anchoring catalyst for high‐sulfur‐loading Li–S batteries, bringing them one step closer to practical application.

## 3 Conclusions

In summary, we have addressed the critical issues of polysulfide shuttling and sluggish redox kinetics in Li–S batteries by developing a functional separator coated with thiol‐modified UiO‐66‐SH. The as‐designed UiO‐66‐SH coating integrates strong chemical adsorption of polysulfides, efficient catalytic conversion, and uniform Li^+^ flux regulation into a single multifunctional layer. Systematic electrochemical and post‐mortem characterizations confirm that the functional separator effectively suppresses the shuttle effect, accelerates redox kinetics, and inhibits lithium dendrite growth. As a result, the Li–S cell delivers outstanding long‐term cycling stability, excellent rate capability, and reliable performance under high sulfur loading, demonstrating marked superiority over pristine PP and unmodified UiO‐66 separators. This work establishes a rational strategy to transform a conventional separator into a multifunctional platform by combining coordinatively unsaturated metal sites with thiol groups. The approach is generalizable to other MOF‐based systems and offers a blueprint for simultaneously suppressing polysulfide shuttling, accelerating sulfur conversion, and stabilizing the lithium anode, paving the way toward practical high‐energy Li–S batteries.

## 4 Experimental Section

### 4.1 Preparation of UiO‐66‐SH, Composite Separator and Electrode

#### 4.1.1 Chemicals and Materials

All chemicals and solvents were used as received without further purification. Zirconium chloride (ZrCl_4_) and 2,4‐mercaptoterephthalic acid (H_2_BDC‐SH) were purchased from Tokyo Chemical Industry and Tianjin Xiensi Biochemical Technology Co., Ltd., respectively. Polyvinylidene fluoride (PVDF), N‐methyl‐2‐pyrrolidone (NMP) and N, N‐dimethylformamide (DMF) were obtained from Shanghai Aladdin Biochemical Technology Co., Ltd. Concentrated hydrochloric acid (35% HCl) was purchased from Sinopharm Chemical Reagent Co., Ltd. CR2032 coin cell cases, PP separators (Celgard 2500), super P, Al foil and Cu foil were supplied by Guangdong Canrd New Energy Technology Co., Ltd. Metallic Li foil was purchased by China Energy Lithium Co., Ltd. The electrolyte (2% lithium nitrate (LiNO_3_) and 1 m LiTFSi in DOL: DME = 1:1 (v/v)) was purchased from Suzhou Duoduo Chemical Technology Co., Ltd.

#### 4.1.2 Synthesis of UiO‐66‐SH/UiO‐66

ZrCl_4_ (0.56 g, 2.4 mmol), H_2_BDC‐SH (0.46 g, 2 mmol)/H_2_BDC (0.33 g, 2 mmol), and 35% HCl (0.574 mL, 5 mmol) were added to 20 mL of DMF. The mixture was ultrasonicated for 30 min, transferred into a 100 mL Teflon‐lined autoclave, and heated at 100 °C for 20 h. After cooling to room temperature, the product was washed three times with DMF (centrifugation at 10 000 rpm for 5 min each time). The solvent was then exchanged with methanol (Aladdin) by soaking for 24 h followed by centrifugation; this exchange process was repeated three times. The resulting solid was dried in a vacuum oven at 80 °C for 10 h to obtain pure UiO‐66‐SH/UiO‐66 powder, which was further activated at 150 °C for 24 h under vacuum prior to use.

#### 4.1.3 Preparation of UiO‐66‐SH/PP(UiO‐66‐SH/PP) Separator

700 mg of activated UiO‐66‐SH (700 mg of activated UiO‐66), 50 mg of PVDF, and 2 mL of NMP were mixed and stirred at 600 rpm for 12 h to form a homogeneous slurry. The slurry was coated onto a 25 μm PP separator using an automatic coater with a doctor blade gap of 10 μm. The uncoated 25 μm PP separator served as the baseline separator.

#### 4.1.4 Preparation of Sulfur Carbon Composites

Mesoporous carbon Keijenblack (KB) and activated carbon (AC), used as carbon hosts for sulfur, were purchased from XFNANO Materials Technology Co., Ltd. Sulfur carbon composites with 70 wt% sulfur loading (S@KB (70%) and S@AC (70%)) were prepared by a melt diffusion method: carbon materials were thoroughly mixed with sulfur powder and heated at 155 °C for 12 h. Sulfur cathodes were fabricated by mixing the sulfur carbon composite, Super P conductive carbon, and PVDF binder in a mass ratio of 8:1:1 in NMP to form a uniform slurry. The slurry was coated onto carbon‐coated aluminum foil, dried at 60 °C for 12 h, and punched into 10 mm diameter disks for cell assembly.

### 4.2 Electrochemical Tests

CR2032‐type coin cells were assembled to evaluate electrochemical performance. The cells consisted of the prepared sulfur cathode, lithium–metal anode, and PP separator (or modified separators). All tests were carried out at 25 °C. Galvanostatic charge–discharge cycling was performed on a Neware battery test system. Each Li–S coin cell was filled with 60 μL of electrolyte (2% LiNO_3_ and 1 m LiTFSI dissolved in a DOL/DME solution with a volume ratio of 1:1) to ensure sufficient electrode wetting, and the voltage window was set to 1.7–2.8 V. For Li|Li symmetric cells (CR2032‐type), two identical lithium electrodes and a separator were used. The cells were cycled at current densities of 0.5 and 1 mA cm^−2^ with areal capacities of 0.5 and 1 mAh cm^−2^, respectively. Li–Cu cells were assembled using a metallic lithium electrode, a metallic copper electrode, and a Celgard 2500 separator. The cells were tested at a current density of 0.5 mA cm^−2^ with an areal capacity of 2 mAh cm^−2^. Cyclic voltammetry (CV) measurements were conducted on a Biologic VSP‐300 electrochemical workstation within voltage ranges of 1.7–2.8 V (for Li–S cells) and −1 to 1 V (for symmetric cells), at a scan rate of 0.1 and 1–9 mV s^−1^. Li–S batteries assembled with different separators were subjected to one charge–discharge cycle at 0.05 C, and then EIS was measured in the frequency range of 0.01 Hz–1 MHz with a perturbation amplitude of 10 mV. The tests were conducted on a Bio‐Logic VSP‐300 electrochemical workstation. The impedance data were processed using an open‐source MATLAB‐based tool (DRTtools) to derive the distribution of relaxation times (DRT) spectra. A nonlinear least‐squares method combined with Tikhonov regularization was employed to fit the experimental data, with Gaussian basis functions used for discretization. Both real and imaginary parts of the EIS data were included in the fitting, while inductive contributions were excluded. Tikhonov regularization was implemented with second‐order derivative parameters, a regularization parameter of 10^−5^, and a radial basis function with a shape factor of 1.6.

EIS was measured in the frequency range of 0.01 Hz–1 MHz, with a perturbation amplitude of 10 mV. Ionic conductivity measurements were performed using a stainless steel (SS)||separator||SS symmetric coin cell by EIS. The tests were conducted on a Bio‐Logic VSP‐300 electrochemical workstation. The ionic conductivity was calculated according to Equation (1):

(1) $$\sigma =\frac{L}{R_{b}S}$$

where *σ* is the ionic conductivity, *L* is the thickness of the separator, *R*_b_ is the bulk resistance, and *S* is the contact area between the separator and the stainless steel. Note that this ionic conductivity value represents the collective conductivity of the separator and the electrolyte, not the intrinsic conductivity of the electrolyte alone.

The Li^+^ transference number was evaluated using Li||separator||Li symmetric coin cells. A direct current potentiostatic polarization of 10 mV was applied, and EIS was performed in the frequency range of 0.01 Hz–1 MHz with a perturbation amplitude of 10 mV. The lithium‐ion transference number was calculated according to Equation (2).

(2) $$t_{{\mathrm{Li}}^{+}}=\frac{I_{s}(\Delta V-I_{0}{R_{i}}^{0})}{I_{0}(\Delta V-I_{S}{R_{i}}^{S})}$$

where Δ*V* is the polarization voltage across the battery, *I*_0_ and *I*_s_ are the initial current and steady‐state current, respectively, and *R*_i_^0^ and *R*_i_^S^ are the initial impedance and steady‐state impedance, respectively.

### 4.3 Material Characterization

X‐ray diffraction (XRD) patterns were collected on a Rigaku SmartLAB SE diffractometer with Cu Kα radiation (*λ* = 1.5406 Å). Data were recorded in the 2*θ* range of 5°–50° with a step size of 0.01° and a scanning speed of 5° min^−1^. The surface morphology of cycled lithium metal was observed using a Hitachi SU‐8200 scanning electron microscope. Li|Li symmetric cells were disassembled in an argon‐filled glove box. The recovered electrodes were thoroughly rinsed with dimethoxyethane (DME), dried under vacuum, and transferred to airtight sample holders for EDS analysis under argon protection. Fourier transform infrared (FTIR) spectroscopy was employed to analyze the surface functional groups of the samples. The measurements were performed on a Thermo Fisher Scientific Nicolet iS50 FTIR spectrometer over a wavenumber range of 4000–400 cm^−1^. The specific surface area and pore structure were characterized by N_2_ adsorption–desorption isotherms using an Anton Paar Autosorb 6100 high‐vacuum gas adsorption analyzer. The DSC experiment was conducted over a temperature range of 40–200 °C at a heating rate of 10 °C min^−1^. The instrument used was a Thermogravimetric Analyzer DE9001 (Shanghai Waters Technologies Co., Ltd.). XPS was performed using a Thermo Fisher ESCALAB Xi+ instrument to analyze the MOF powders. The thermal behavior of MOF powder was investigated using a TA TGA Q500 thermal gravimetric analyzer.

### 4.4 Characterization of the Modified Separator Performance

The wettability of the electrolyte on the separator surface was evaluated by contact angle measurements using a ZR‐SDJ‐B3 contact angle meter (Suzhou Definuo Electronics Co., Ltd.). The mechanical properties of the materials were evaluated by tensile stress–strain testing using a ZQ‐950 LB universal testing machine (Dongguan Zhiqu Precision Instrument Co., Ltd.) at a tensile rate of 10 mm min^−1^. The thermal shrinkage performance of the separator can be used to characterize its thermal stability and safety. In this experiment, thermal shrinkage tests were conducted at 100, 120, 140, and 160 °C using a DHG‐9035A electric blast drying oven. For UV–vis analysis, different separators were soaked in a 1 mm Li_2_S_6_ solution (DOL/DME = 1:1 by volume) for 3 days, and the UV–vis absorption spectra were recorded using a Shimadzu UV‐3600 spectrometer. The shuttle current was tested to quantitatively evaluate the intensity of the polysulfide shuttle effect. Prior to the measurement, the Li–S coin cells were activated by three cycles at 0.05 C. Subsequently, the cells were charged to 2.40 V and switched to potentiostatic mode. Under a constant temperature of 25 °C, potentiostatic discharge was performed at various voltages (e.g., from 2.9 to 2.2 V) for 2 h at each voltage. The current variation was continuously monitored until a steady state was reached. The steady‐state average current was finally taken as the shuttle current value.

The electrolyte uptake was calculated according to the equation:

(3) $$\text{Electrolyte uptake}\left(\%\right)=\frac{\left(W_{t}-W_{0}\right)100\%}{W_{0}}$$

where *W*_0_ and *W* *_t_* are the weight of the separators before and after the immersion in electrolyte.

The porosity was calculated according to the following equation:

(4) $$\text{Porosity}\left(\%\right)=\frac{(W_{t}-W_{0})100\%}{\rho V}$$

where *W*_0_ and *W* *_t_* represent the weight of dry separators and after n‐butanol absorption, *ρ* is the density of n‐butanol, and *V* is the volume of the separators.

### 4.5 Visualization Experiment of the Shuttle Effect

Li_2_S_6_ solution was prepared by weighing 16.03 mg of sulfur and 4.59 mg of lithium sulfide (Li_2_S) at a molar ratio of 5:1. The solid mixture was dissolved in 10 mL of a mixed solvent composed of equal volumes of 1,3‐dioxolane and dimethoxyethane. The reaction vessel was sealed and stirred continuously at 60 °C for 20 h to obtain 10 mL of a 1 mmol/L Li_2_S_6_ solution. For the shuttle‐effect inhibition test, different separators were fixed in the middle of an H‐type electrolytic cell. Then, 6 mL of the same mixed solvent (equal volumes of DOL and DME) was added to the left compartment, and 6 mL of a 0.1 mmol/L Li_2_S_6_ solution was added to the right compartment. The H‐type cell was sealed and left to stand for the experiment.

### 4.6 Li_2_S Nucleation and Dissolution Experiments

Li_2_S_8_ electrolyte was prepared by reacting sulfur and Li_2_S in a molar ratio of 7:1 in tetraethylene glycol dimethyl ether (TEGDME). For cell assembly, the MOF powder was mixed with Keijen black (KB) and PVDF in a weight ratio of 6:2:2, coated onto carbon‐coated aluminum foil, and dried in a vacuum oven. The coated foil was punched into 12 mm‐diameter disks to serve as the working electrode. 20 μL of 0.2 m Li_2_S_8_ solution was used as the electrolyte, with lithium foil as the counter electrode and Celgard 2500 as the separator. For the nucleation test, cells were first discharged to 2.06 V at a constant current of 0.112 mA, followed by a potentiostatic discharge at 2.05 V until the current dropped below 10^−5^ A. For the dissolution test, cells were first galvanostatically discharged at 0.1 mA to 1.70 V to fully convert LiPSs into solid Li_2_S, and then held potentiostatically at 2.40 V until the charging current fell below 10^−5^ A, ensuring complete dissolution of Li_2_S. This work was carried out using the Neware (MIHW‐200‐160 CH‐B) battery test system (Shenzhen Neware Electronics Co., Ltd.).

### 4.7 Li_2_S_6_ Symmetric Cells

Li_2_S_6_ electrolyte was prepared by reacting sulfur and Li_2_S in a molar ratio of 5:1 in a mixed solvent of 1,3‐dioxolane and dimethoxyethane (volume ratio 1:1). The working electrode was prepared by coating the MOF powder (mixed with PVDF in a 9:1 weight ratio) onto carbon‐coated aluminum foil and drying under vacuum. The coated foil was punched into 12 mm disks. 25 μL of 0.5 m Li_2_S_6_ solution was used as the electrolyte, with Celgard 2500 as the separator. CV measurements were performed between −1 and 1 V at scan rates ranging from 1 to 9 mV s^−1^. This work was carried out using the Neware (MIHW‐200‐160 CH‐B) battery test system (Shenzhen Neware Electronics Co., Ltd.).

## Author Contributions

M.C. and Y.L. conceived the idea and designed the experiments. M.C. synthesized the materials, fabricated the composite separator, characterized the structure, conducted the electrochemical performance, wrote the draft manuscript. Y.L. helped to test the electrochemical performance. X.Z., Y.S., F.S., X.G., Z.L., and C.X. helped to analyze the electrochemical data. Y.P., Y.F., K.X., and Y.L. discussed the results and contributed to the data analysis. Y.L. revised the paper and supervised this work.

## Funding

This work was supported by National Natural Science Foundation of China (52433011, 52203347); opening Foundation of State Key Laboratory of Organic‐Inorganic Composites, Beijing University of Chemical Technology (oic‐202401006); Fundamental Research Funds for the Central Universities of Beijing University of Chemical Technology (PY2602).

## Conflicts of Interest

The authors declare no conflicts of interest.

## Supporting information

Supplementary Material

## Data Availability

The data that support the findings of this study are available from the corresponding author upon reasonable request.
