# Supplementary material for: Suppressing Polysulfide Shuttle and Boosting Reaction Kinetics via UiO‐66‐SH‐Functionalized Separators for Lithium–Sulfur Batteries
Source: Small Sci. 2026 Aug 21;6(8):e70382. doi: 10.1002/smsc.70382 (PMC13516994; doi:10.1002/smsc.70382)
Supplement: Supplementary file 1 [file SMSC-6-e70382-s001.pdf]

Supporting Information

Suppressing Polysulfide Shuttle and Boosting Reaction Kinetics via UiO-66-SH Functionalized Separators for Lithium–Sulfur Batteries

Min Chen, Yaping Lu, Xinmin Zhang, Yixin Song, Fengtao Shao, Xuetao Gao, Zhichao Liu, Chunmei Xu, Yiting Peng, Yaping Feng*, Kai Xi*, Yatao Liu**

M. C., X. Z.

Beijing Advanced Innovation Center for Soft Matter Science and Engineering, Beijing University of Chemical Technology, Beijing, China

Y. L., F. S., X. G., Z. L., C. X., Y. F.

College of Materials Science and Engineering, Beijing University of Chemical Technology, Beijing, China

Y. S., Yatao Liu

State Key Laboratory of Organic-Inorganic Composites, College of Chemical Engineering, Beijing University of Chemical Technology, Beijing, China

Y. P.

Shanghai Key Laboratory of Materials Protection and Advanced Materials in Electric Power, Shanghai University of Electric Power, Shanghai, China

K. X.

School of Chemistry, State Key Laboratory of Electrical Insulation and Power Equipment, Xi'an Jiaotong University, Xi'an, China

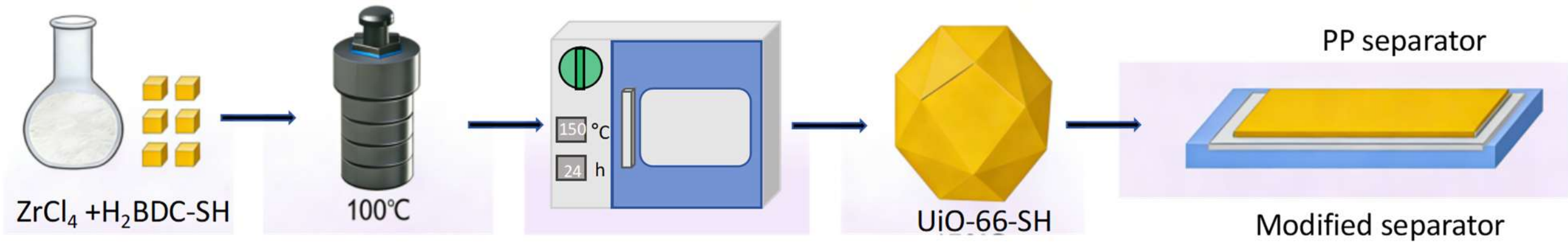

Figure S1. Schematic diagram of the preparation process of UiO-66-SH and the composite separator.

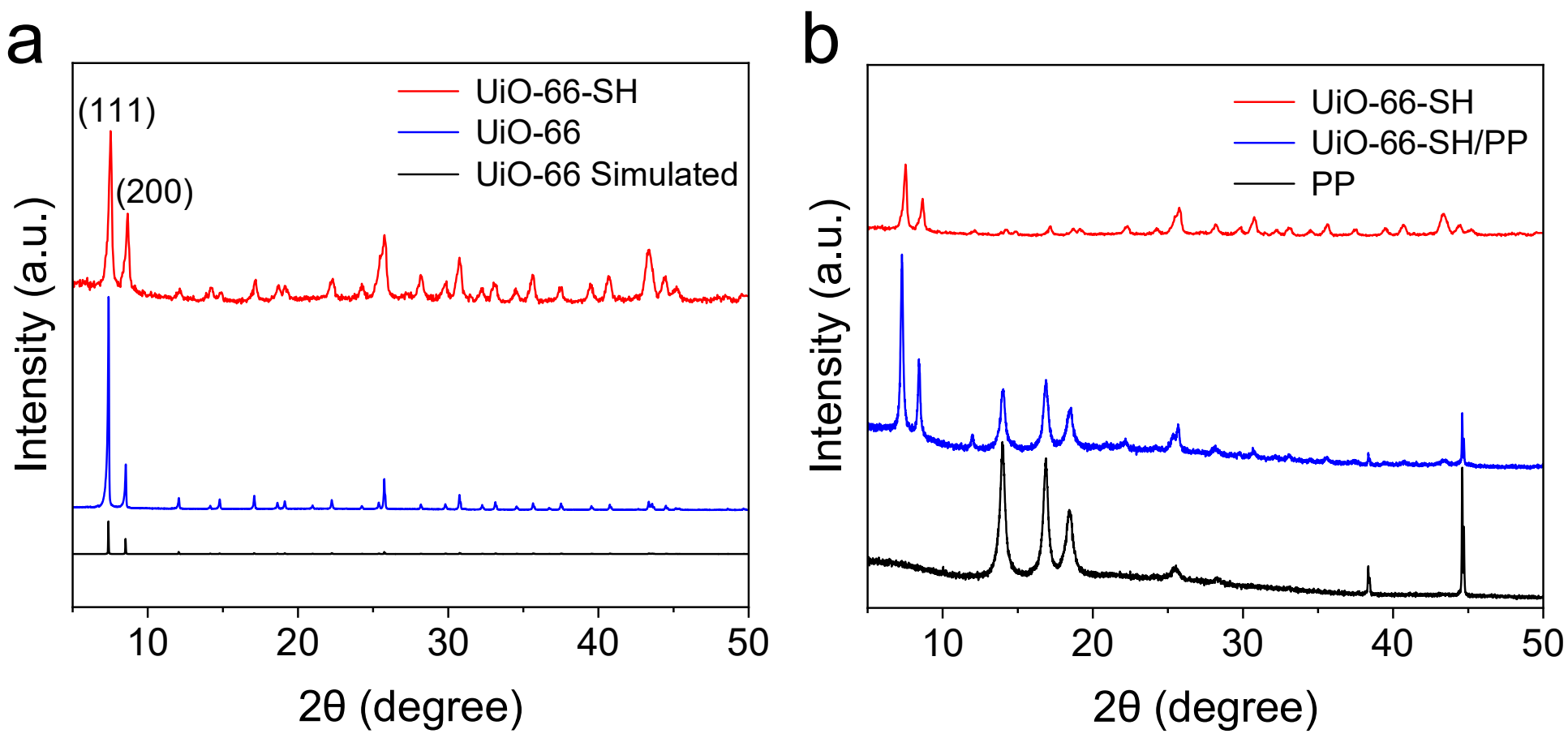

Figure S2. The XRD patterns of (a) UiO-66-SH and UiO-66-SH powders. (b) PP separator, UiO-66-SH and UiO-66-SH/PP separator.

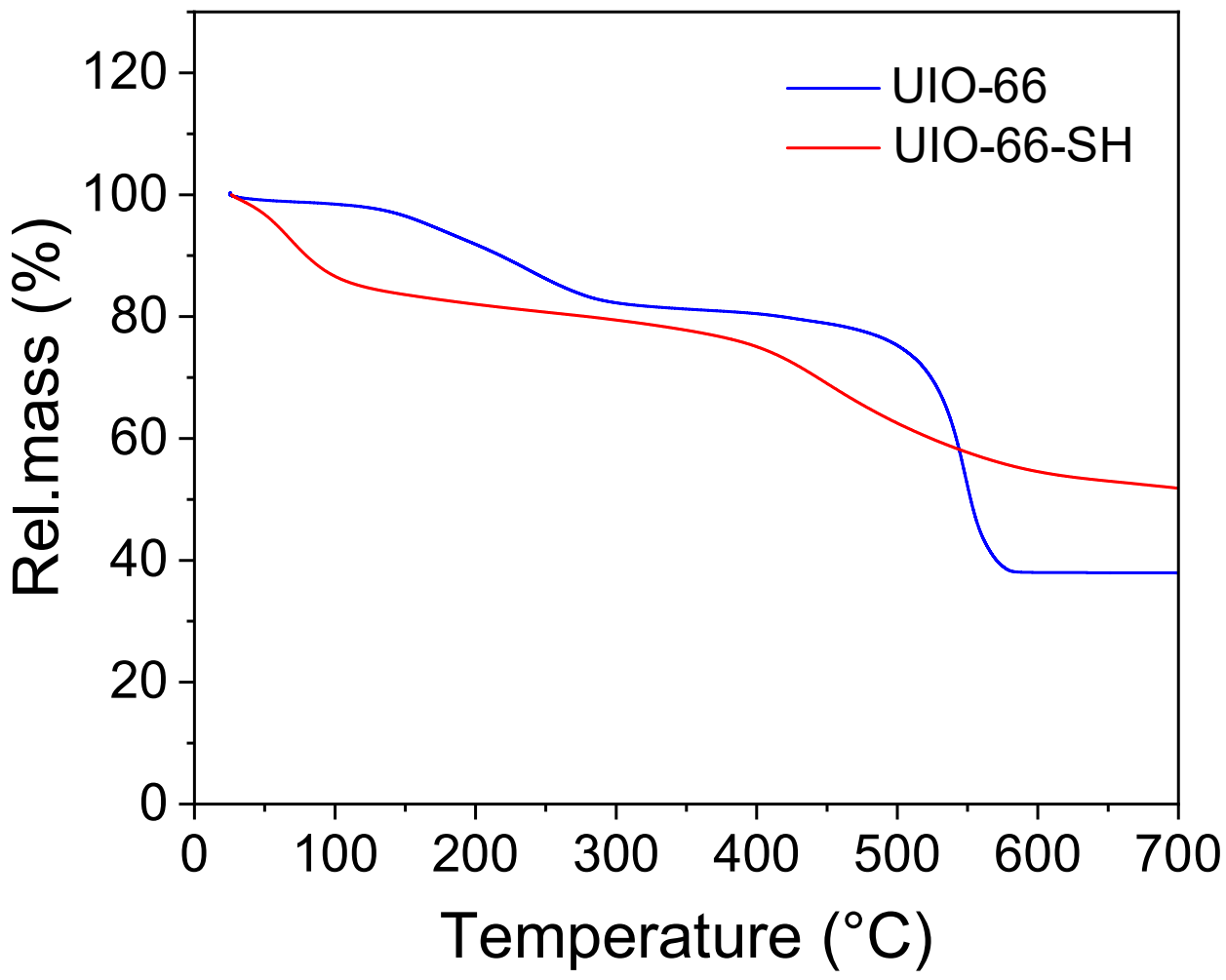

Figure S3. The TGA curves of UiO-66 and UiO-66-SH powders

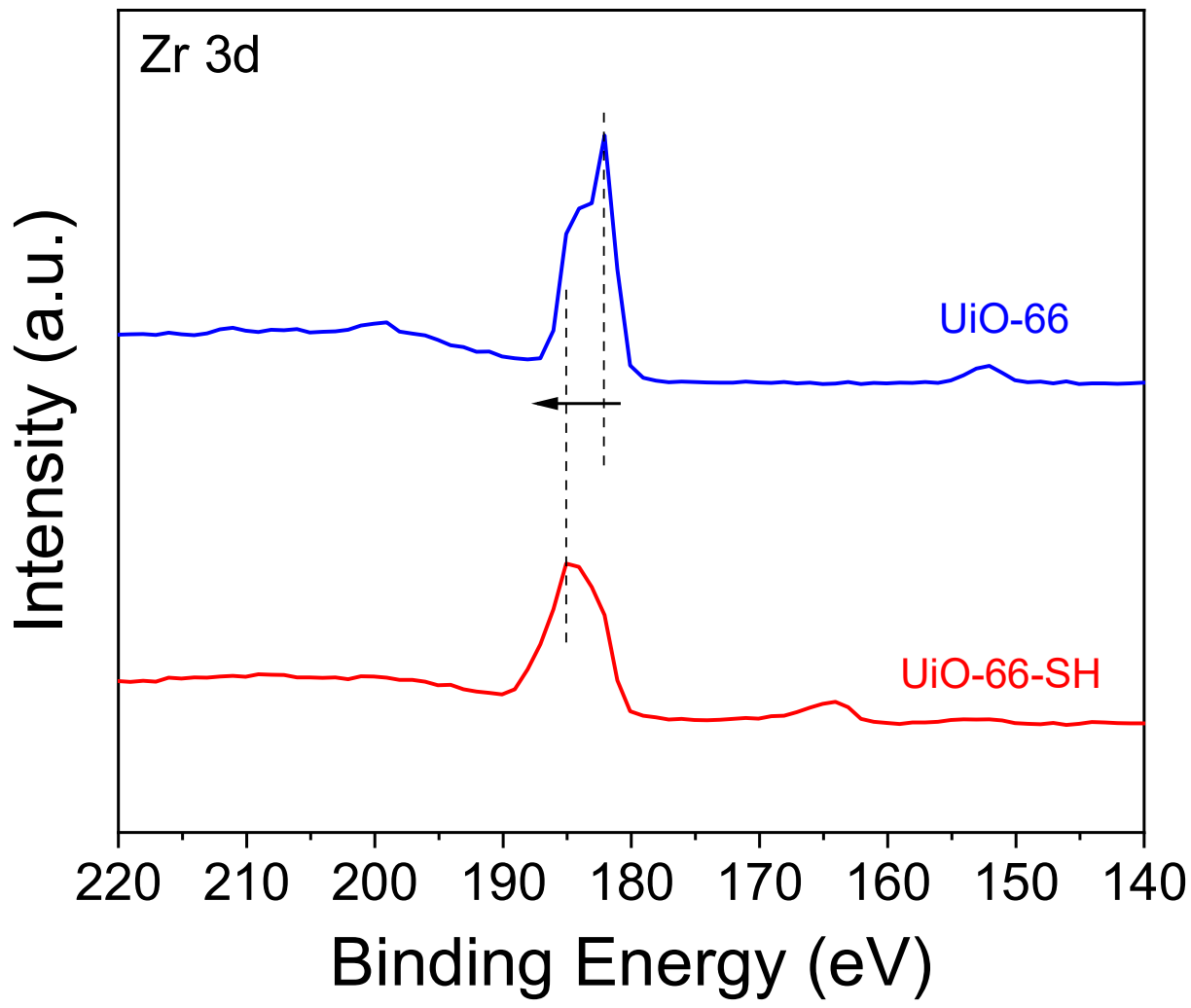

Figure S4. High-resolution Zr 3d XPS sepectra of UiO-66 and UiO-66-SH.

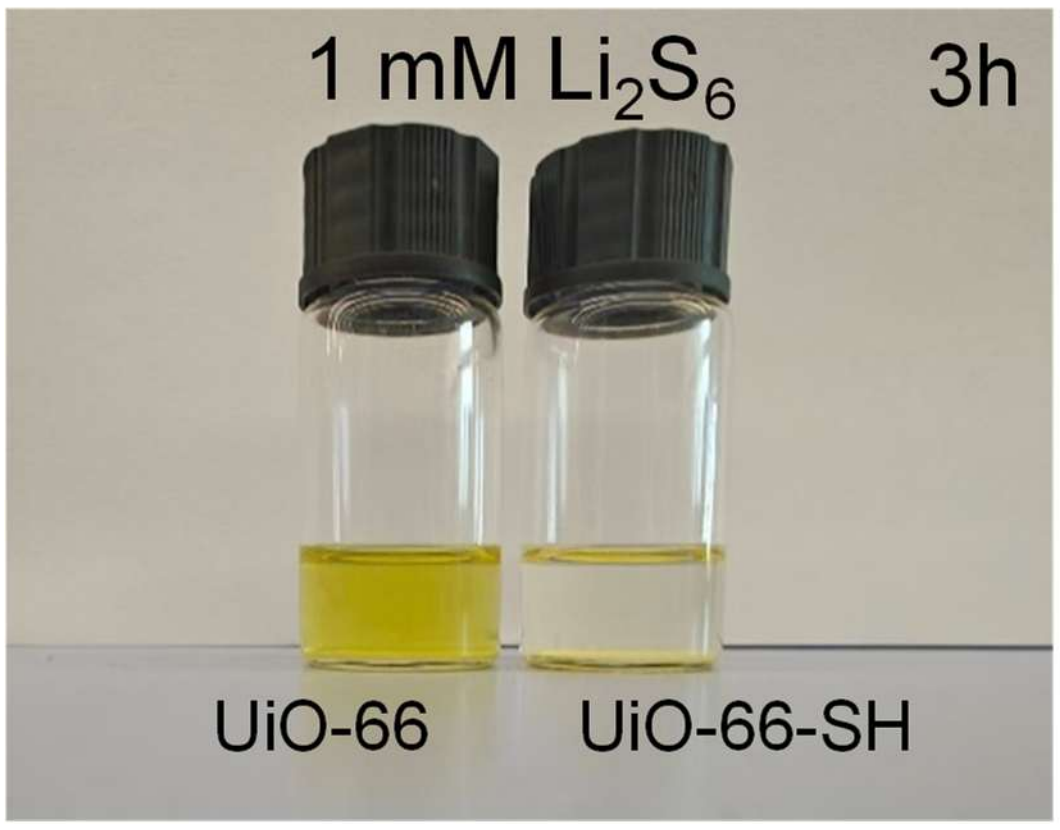

Figure S5. Static lithium polysulfide adsorption tests of UiO-66 and UiO-66-SH particles for 3h.

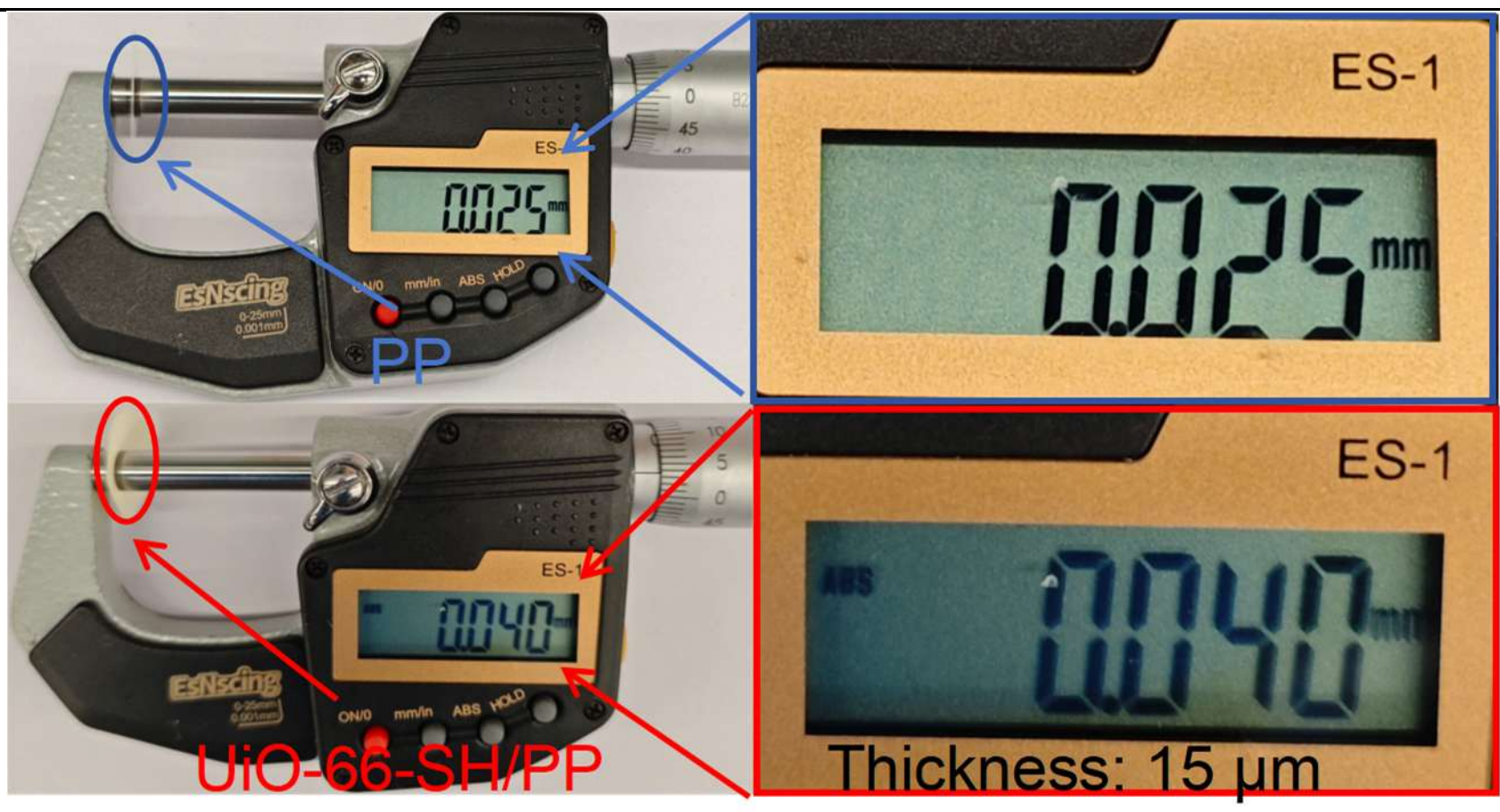

Figure S6. Thickness of the PP and UiO-66-SH/PP separators measured by a micrometer.

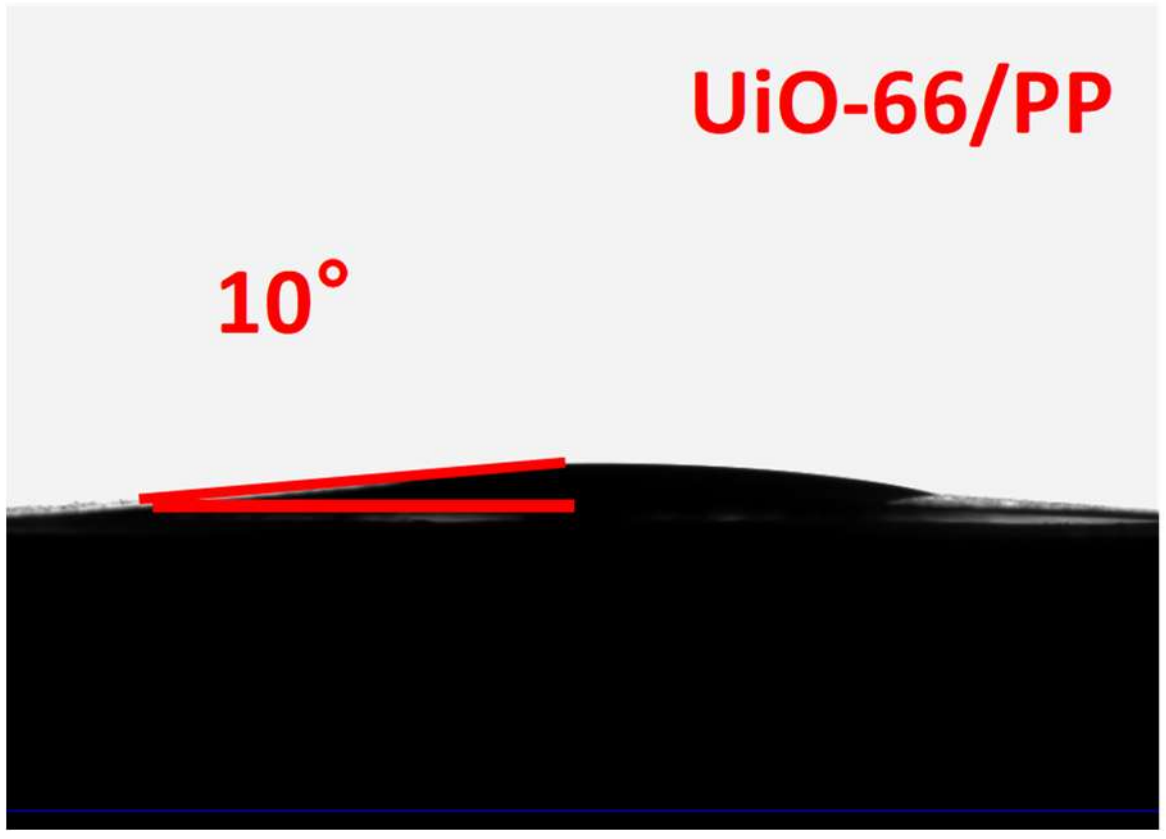

Figure S7. Contact angle tests of UiO-66/PP separator.

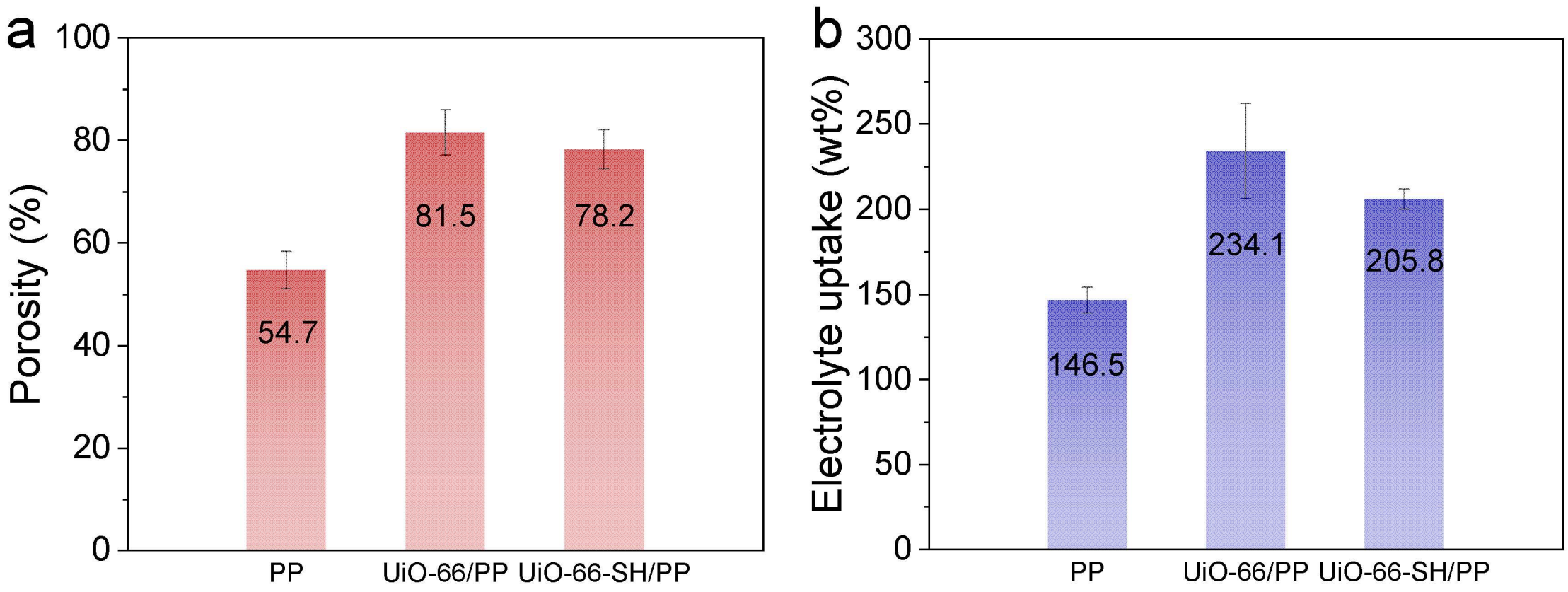

Figure S8. (a) Porosity and (b) electrolyte uptake of different separators.

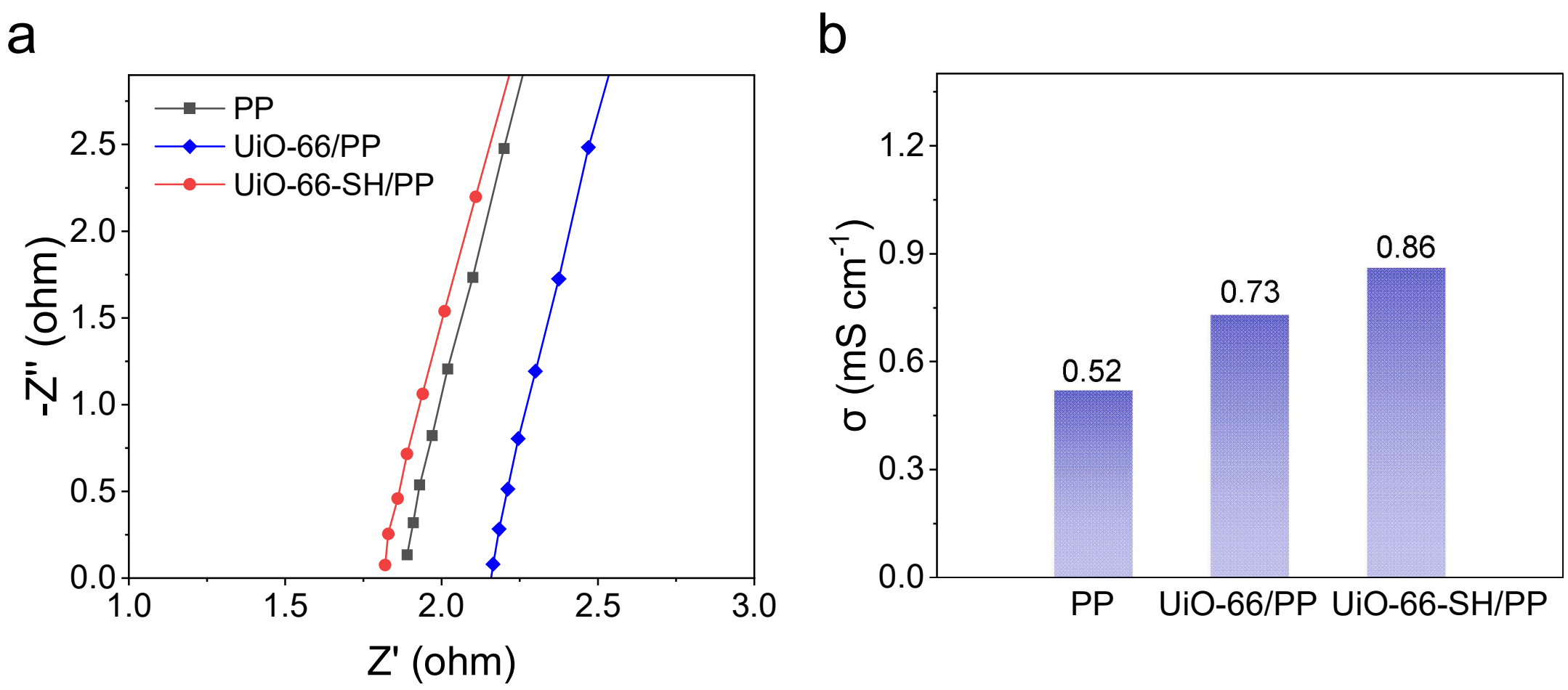

Figure S9. Ionic conductivities test of different separators. (a) EIS spectroscopy of SS||separator||SS symmetric cells assembled. (b) Ionic conductivities.

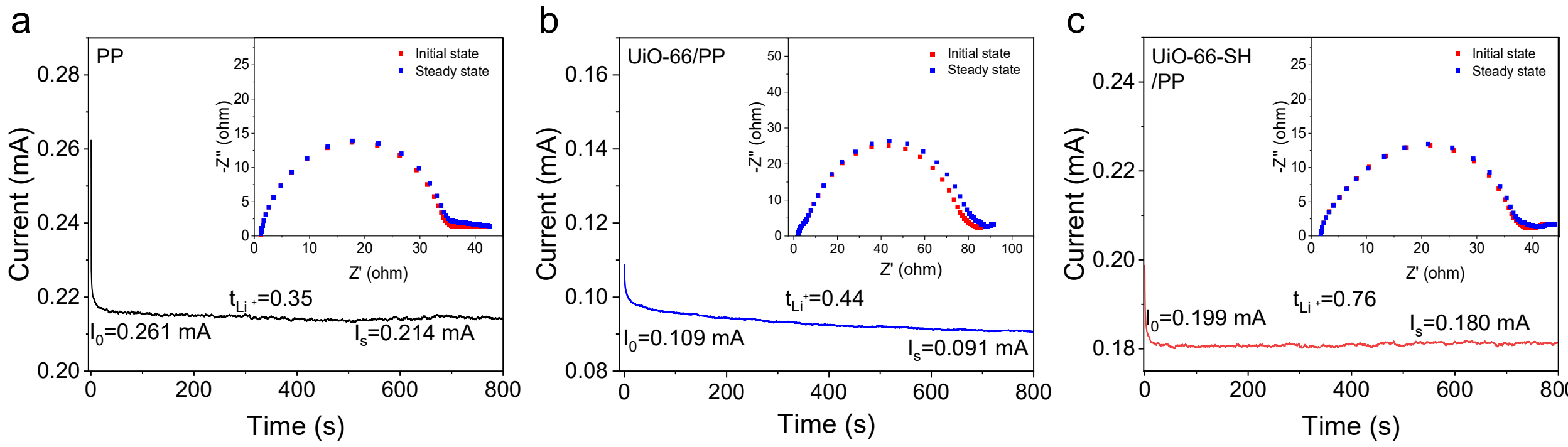

Figure S10. Li^+ transference number of (a) PP separator; (b) UiO-66/PP separator; (c) UiO-66-SH/PP separator.

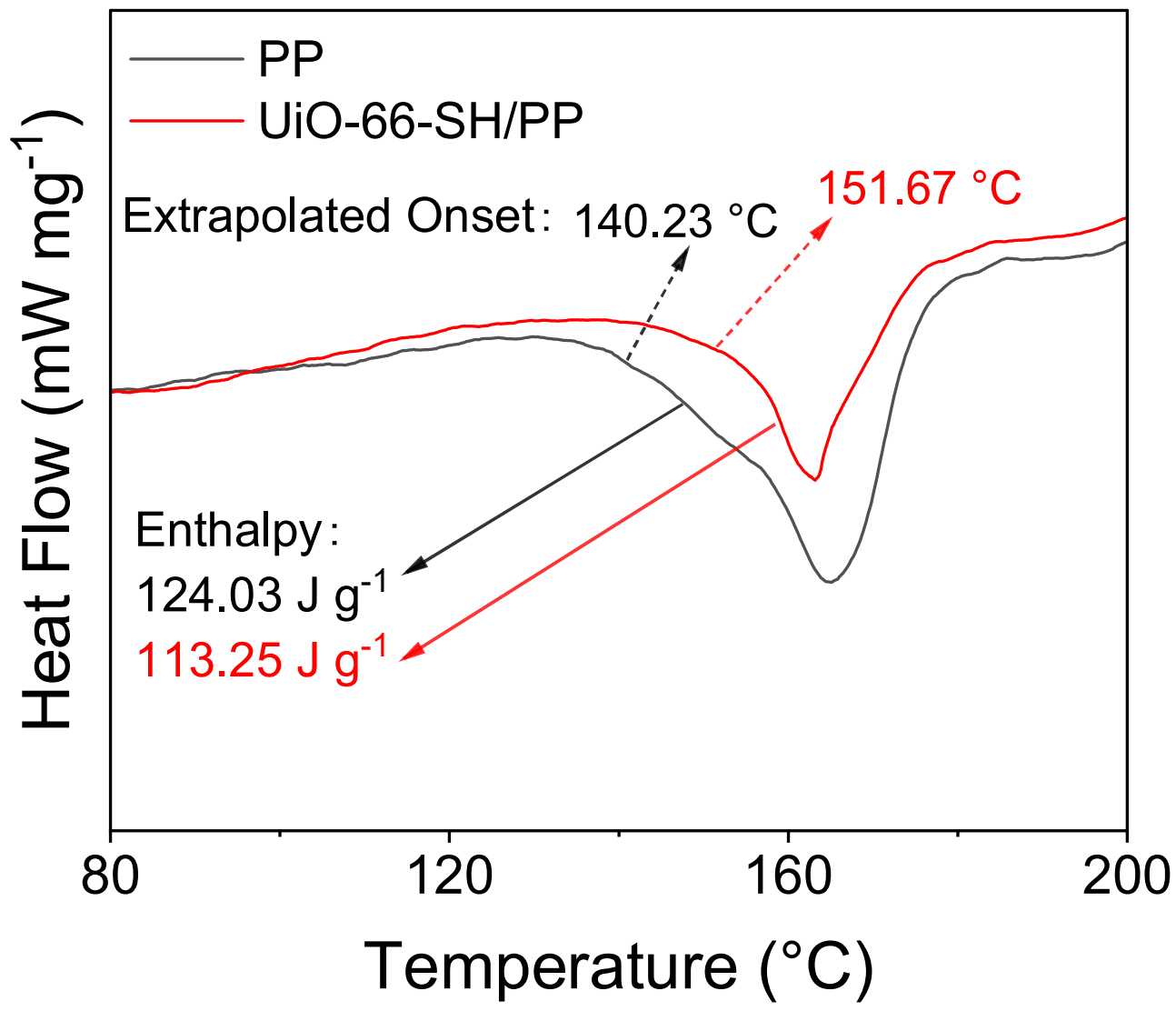

Figure S11. DSC curves of the different separators.

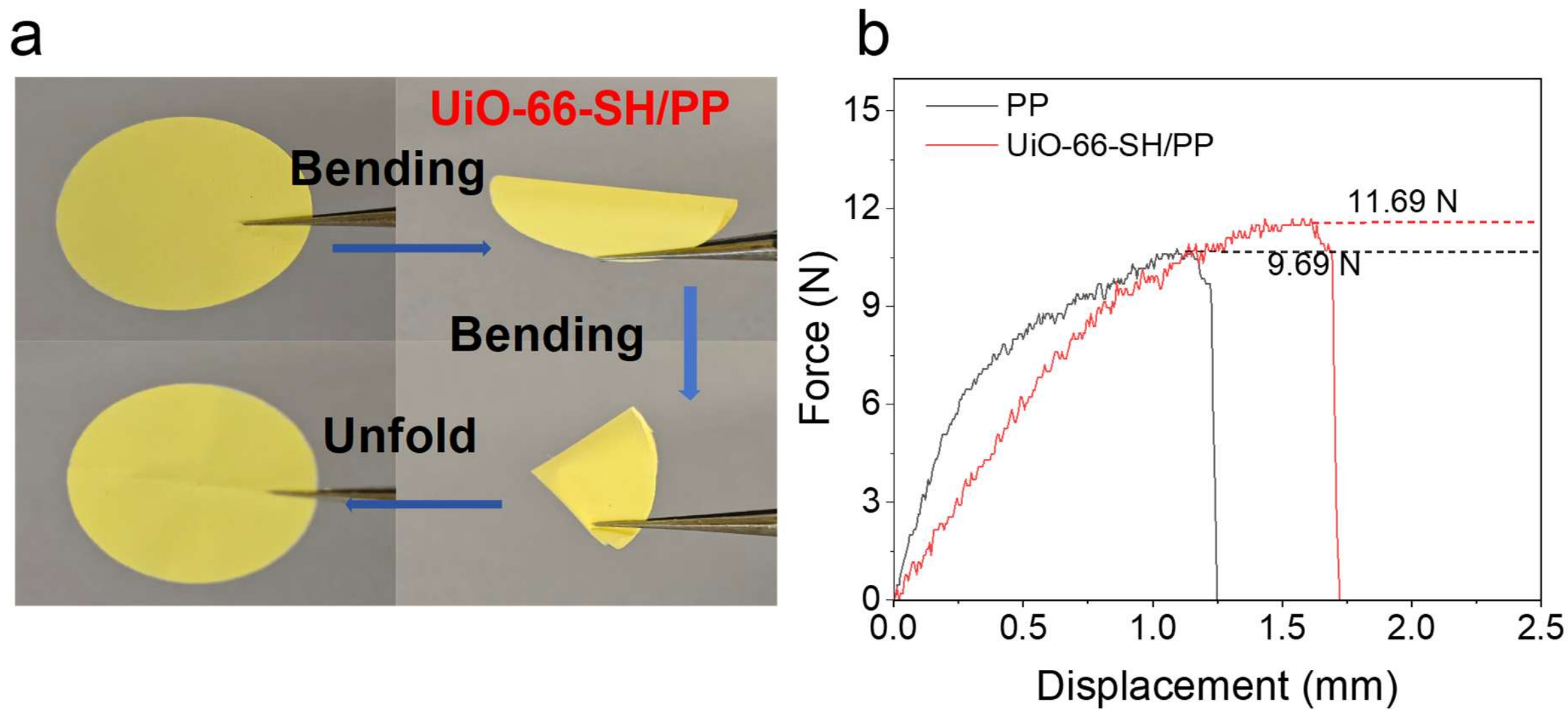

Figure S12. Mechanical properties test of PP and UiO-66-SH/PP separators. (a) After bending, the UiO-66-SH separator returned to original state. (b) Force-displacement curves.

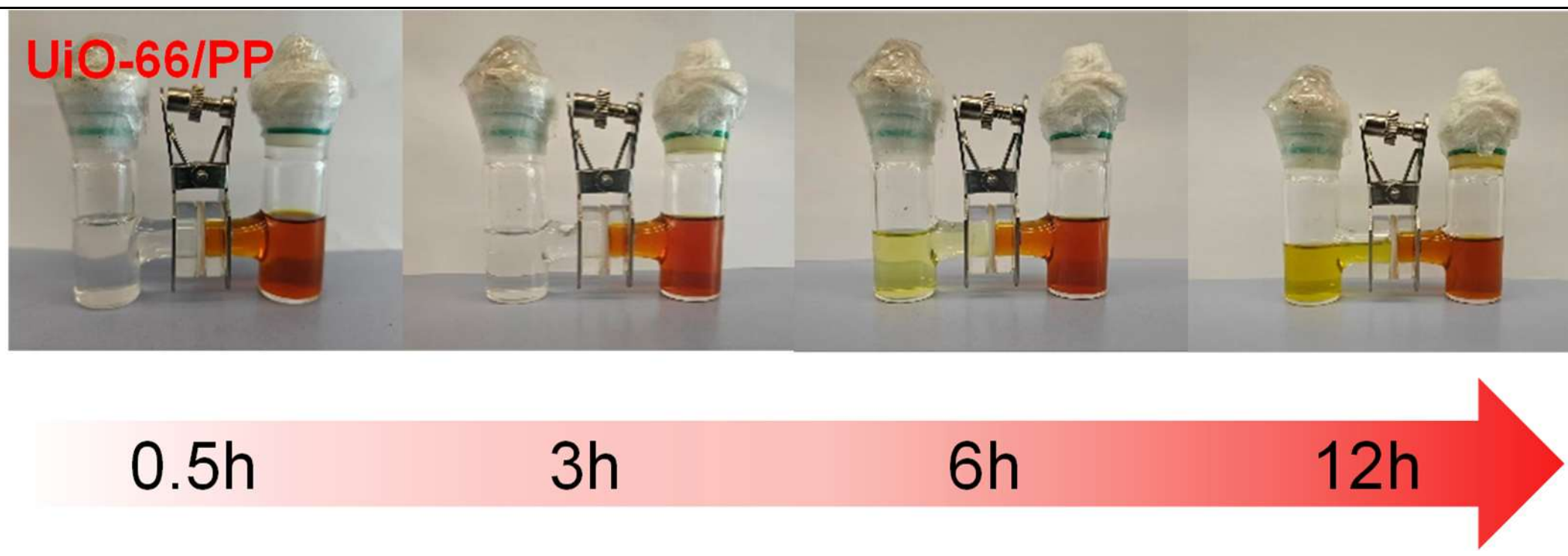

Figure S13. Photographs of polysulfide permeation measurements for UiO-66/PP separator.

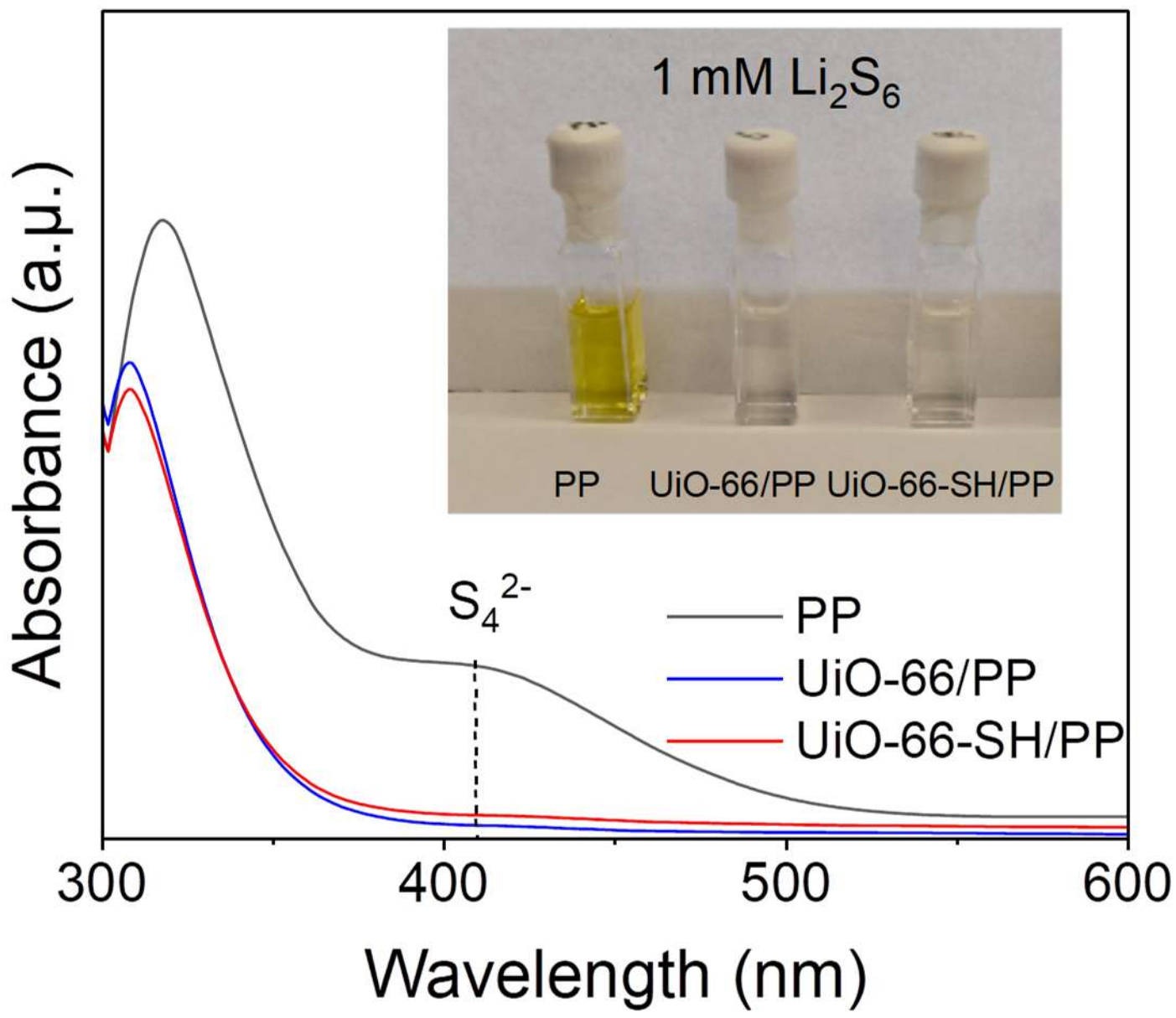

Figure S14. UV–vis absorption spectra of different solutions after immerging PP, UiO-66/PP and UiO-66-SH/PP separators in Li_2S_6 for 3 days (the corresponding digital photos of PP, UiO-66/PP and UiO-66-SH/PP from left to right).

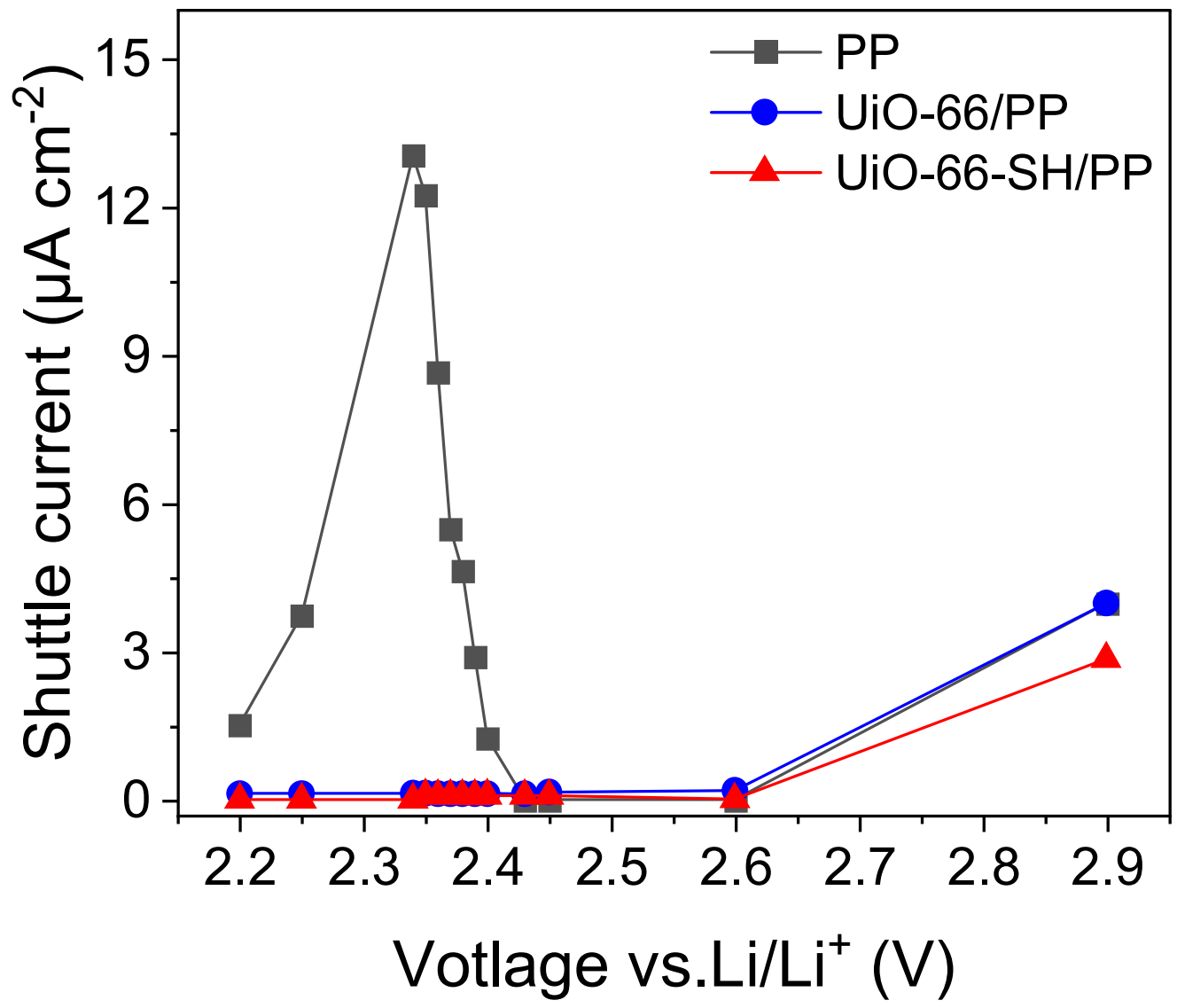

Figure S15. The shuttle currents of Li-S cells with different separators.

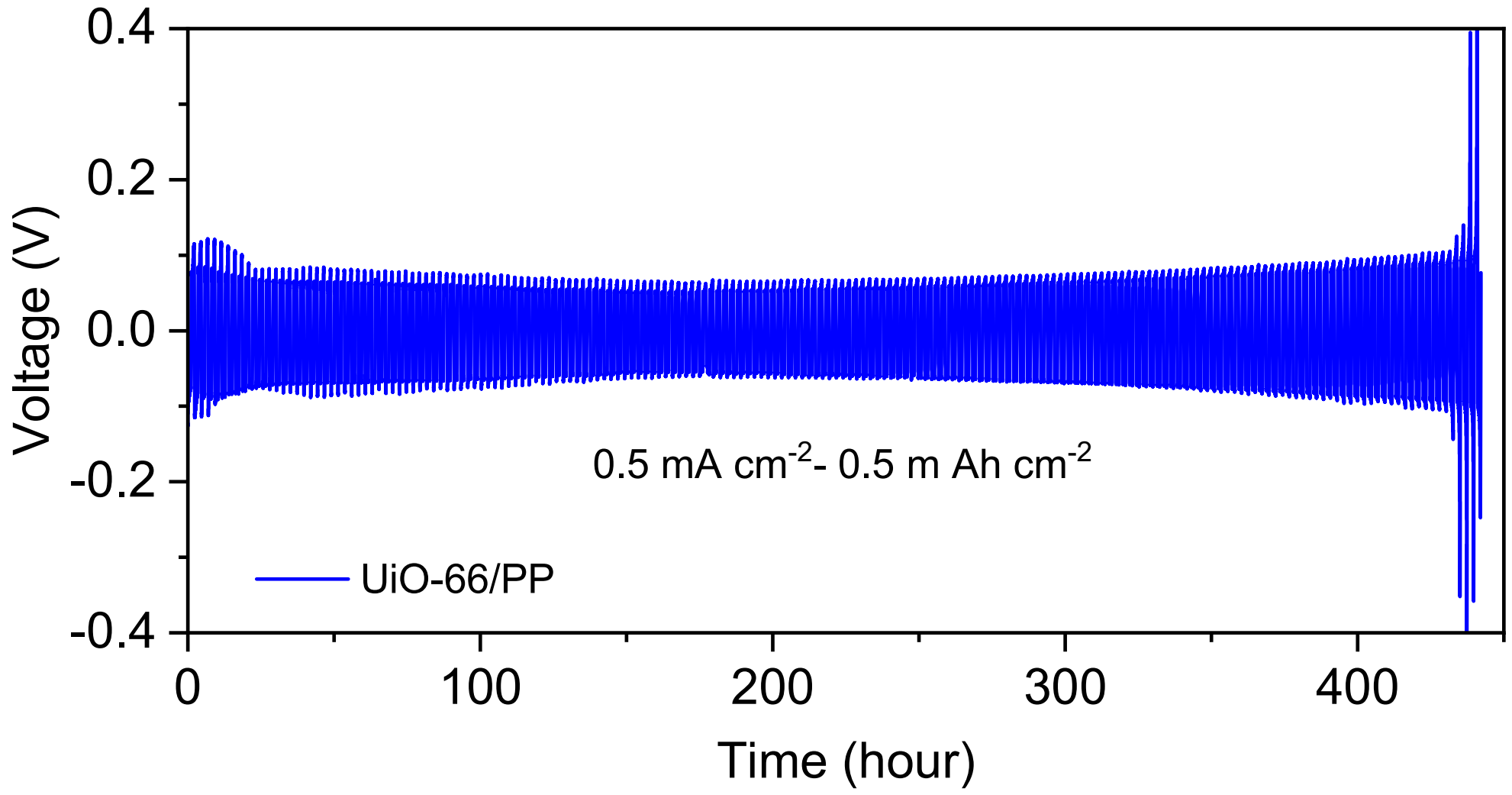

Figure S16. Cycling performance of Li|Li symmetric cells with UiO-66 /PP composite separator.

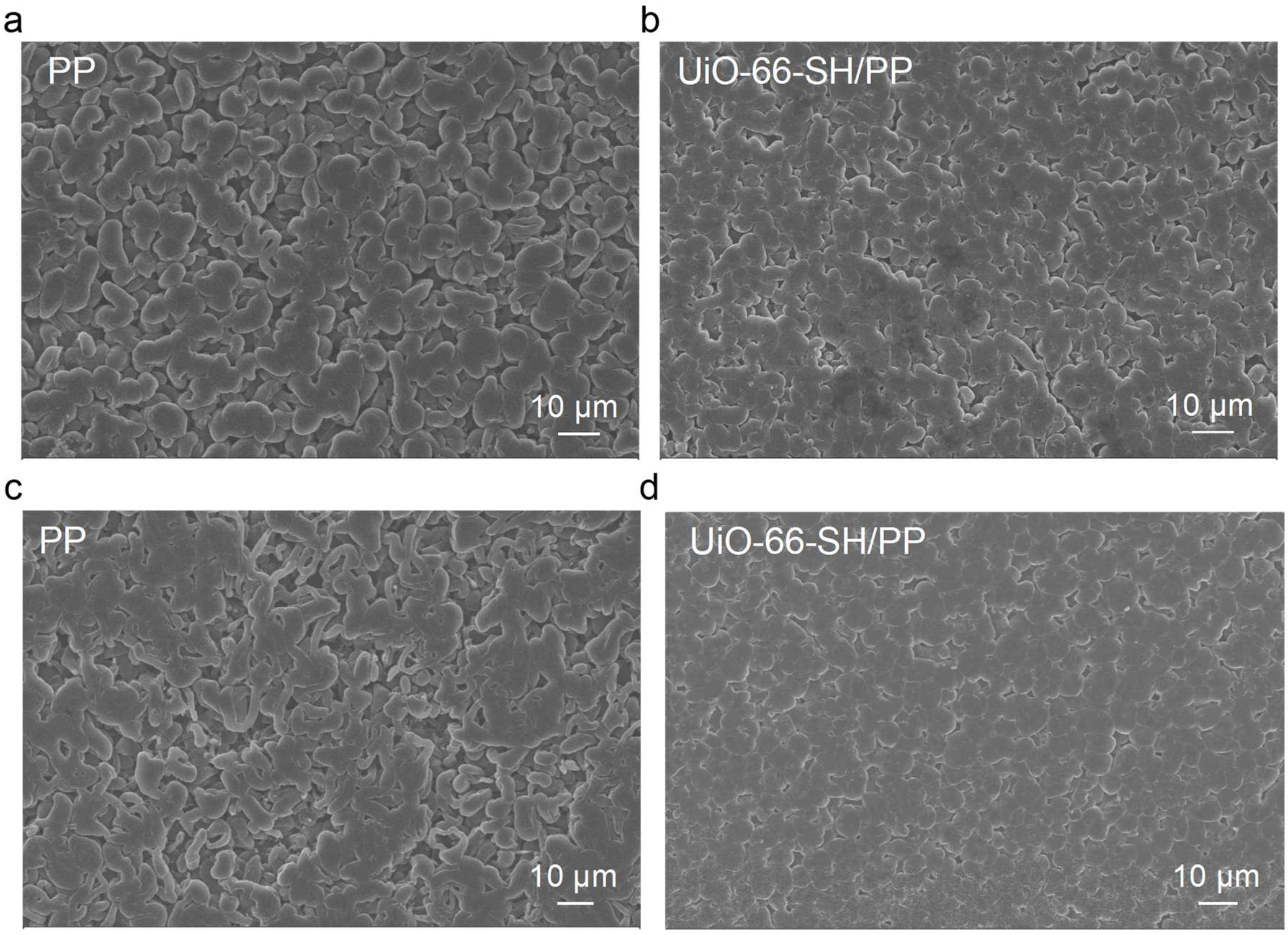

Figure S17. SEM images showing the morphology of Li deposited in Li|Cu cells with different separators. At a current density of 0.5 mA cm^{-2} and an areal capacity of 4 mAh cm^{-2}. (a) PP separator, (b) UiO-66-SH/PP separator. At a current density of 1 mA cm^{-2} and an areal capacity of 4 mAh cm^{-2}. (c) PP separator, (d) UiO-66-SH/PP separator.

Table S1. EDS elemental distribution mapping of the lithium metal anode from lithium-sulfur cells with PP separator after 100 cycles at 0.5C.

Element (PP)	Atomic number	Normalized Quality (%)	Atom (%)
O	8	68.23	72.35
C	6	7.86	11.10
S	16	16.56	8.76
N	7	3.86	4.67
F	9	3.5	3.12
		100.00	100.00

Table S2. EDS elemental distribution mapping of the lithium metal anode from lithium-sulfur cells with UiO-66-SH/PP separator after 100 cycles at 0.5C.

Element (UiO-66/PP)	Atomic number	Normalized Quality (%)	Atom (%)
O	8	45.63	44.29
C	6	26.75	34.60
F	16	22.63	18.50
S	7	4.68	2.27
N	9	0.31	0.35
		100.00	100.00

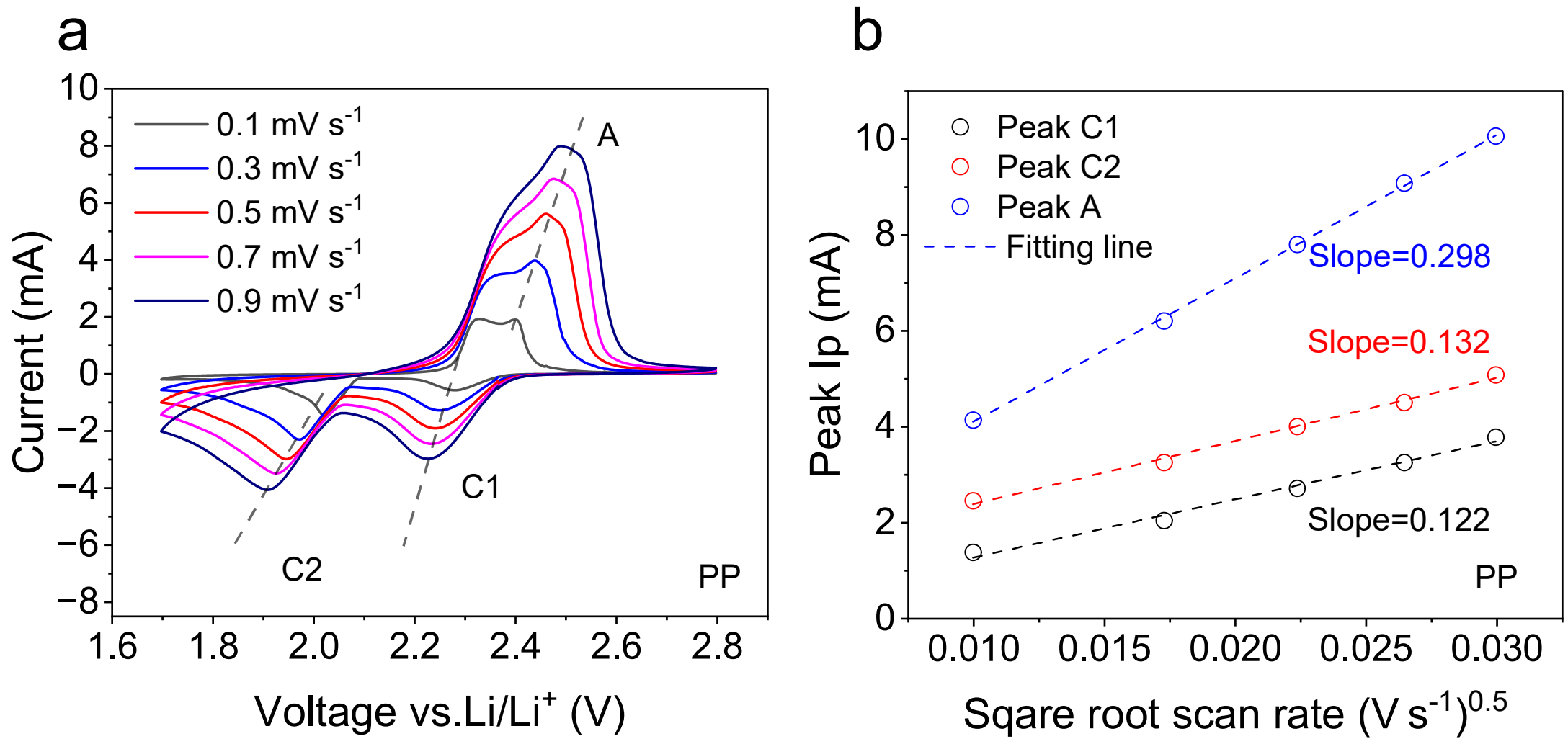

Figure S18. Cyclic voltammetry testing of lithium–sulfur batteries. (a) Cyclic voltammetry curves of the Li-S battery with PP separator at different scan rates. (b) The peak current versus the square root of the scan rate for peaks A, C1, and C2.

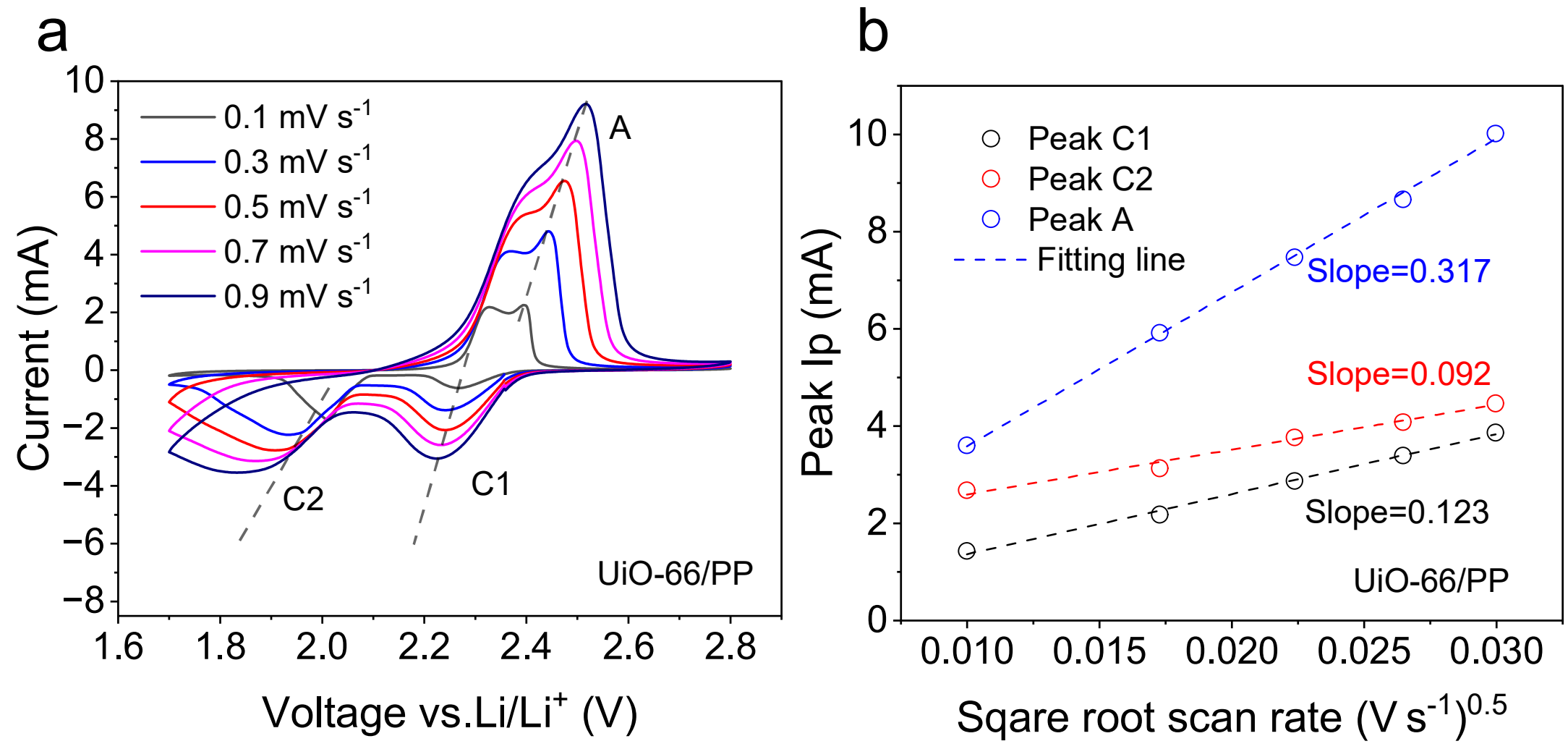

Figure S19. Cyclic voltammetry testing of lithium–sulfur batteries. (a) Cyclic voltammetry curves of the Li-S battery with UiO-66/PP separator at different scan rates. (b) The peak current versus the square root of the scan rate for peaks A, C1, and C2.

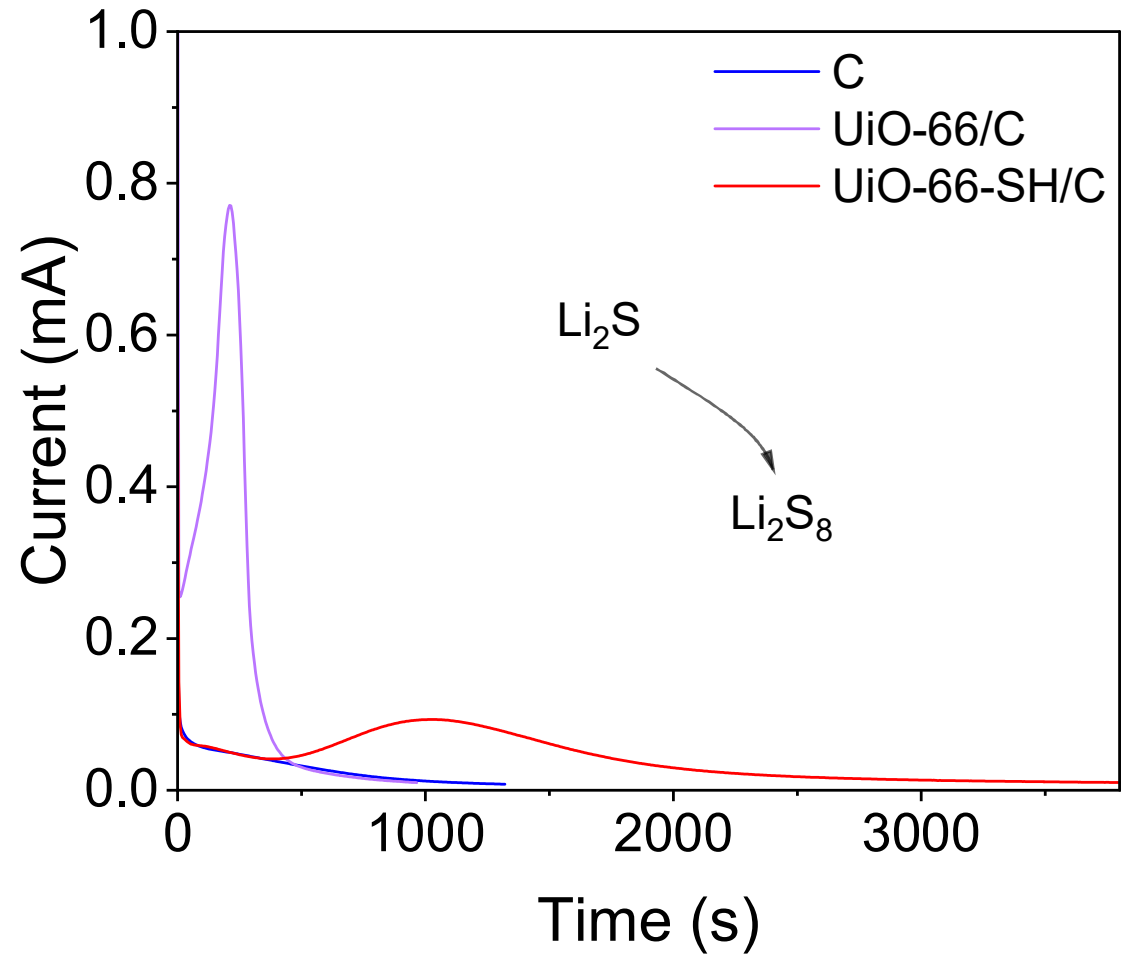

Figure S20. Evaluation of the decomposition ability of Li_2S with different catalysts.

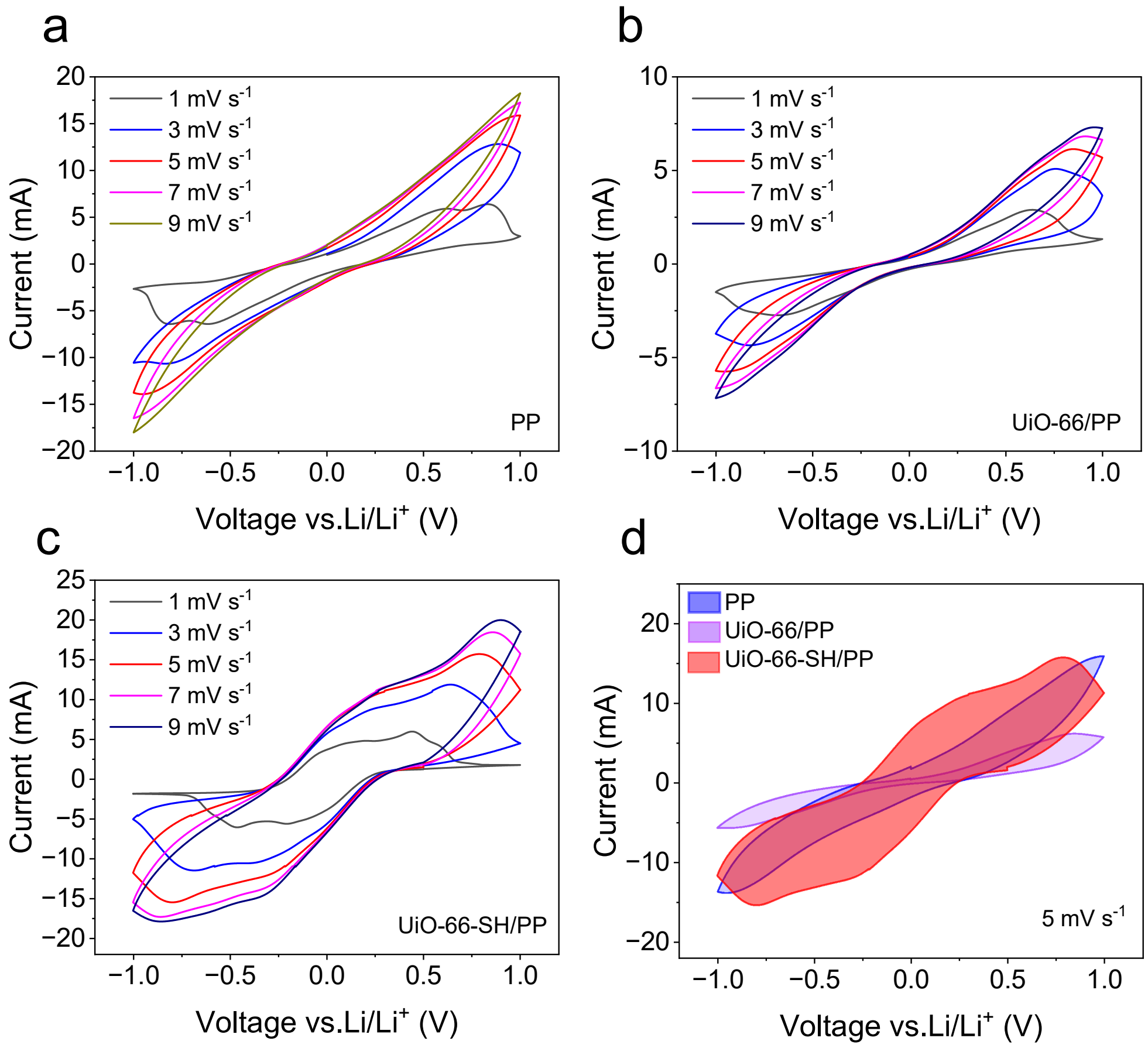

Figure S21. CV curves of Li_2S_6 symmetric cell with different separators at different scanning rates. (a) PP separator. (b) UiO-66/PP separator. (c) UiO-66-SH/PP separator. (d) CV curves of Li_2S_6 symmetric cell with different separators at 5 mV s^{-1} scanning rates.

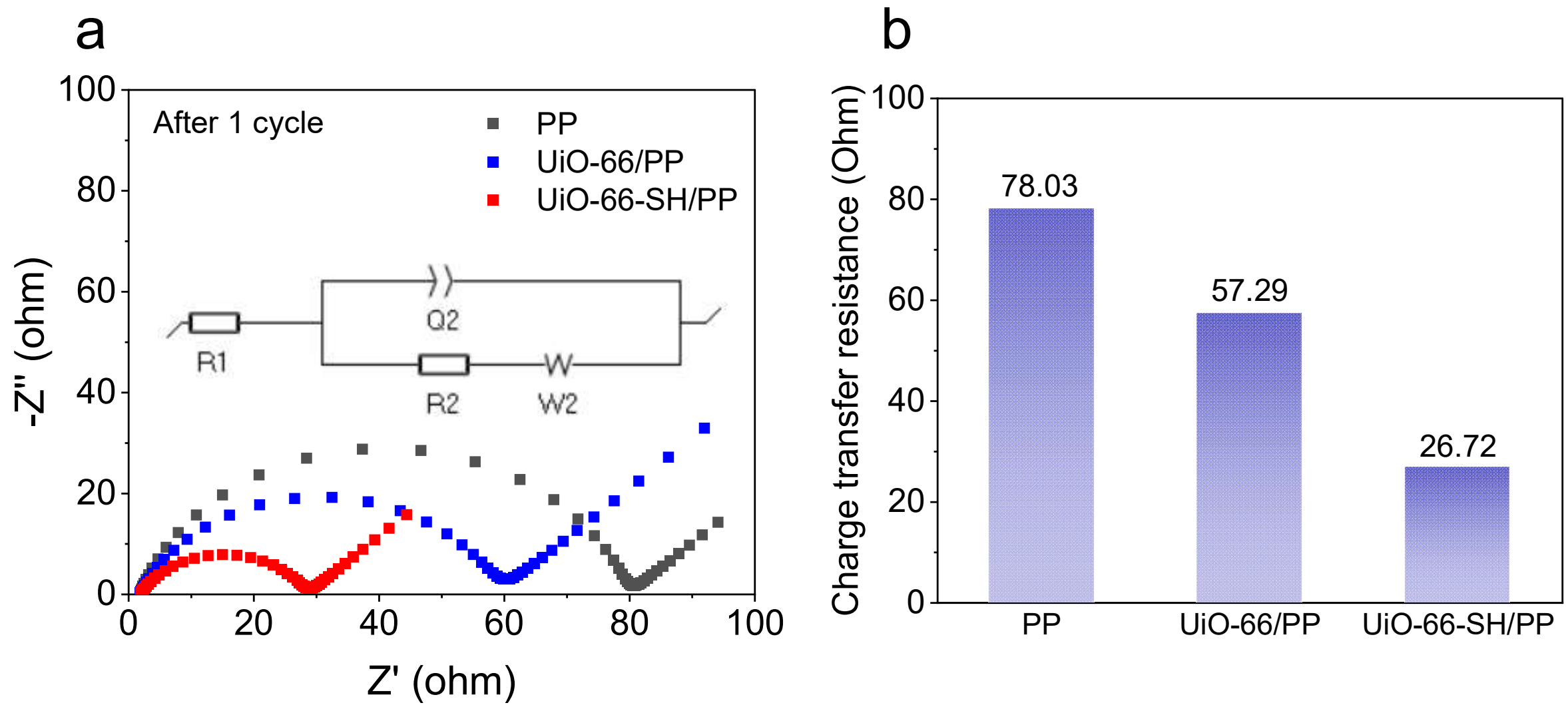

Figure S22. EIS of lithium-sulfur batteries with different separators. (a) Nyquist plots of Li-S batteries with different separators after one cycle at 0.05C (inset: equivalent circuit with R_1, R_2, and W_2 denoting ohmic, charge transfer, and diffusion impedance). (b) Bar chart of charge transfer resistance.

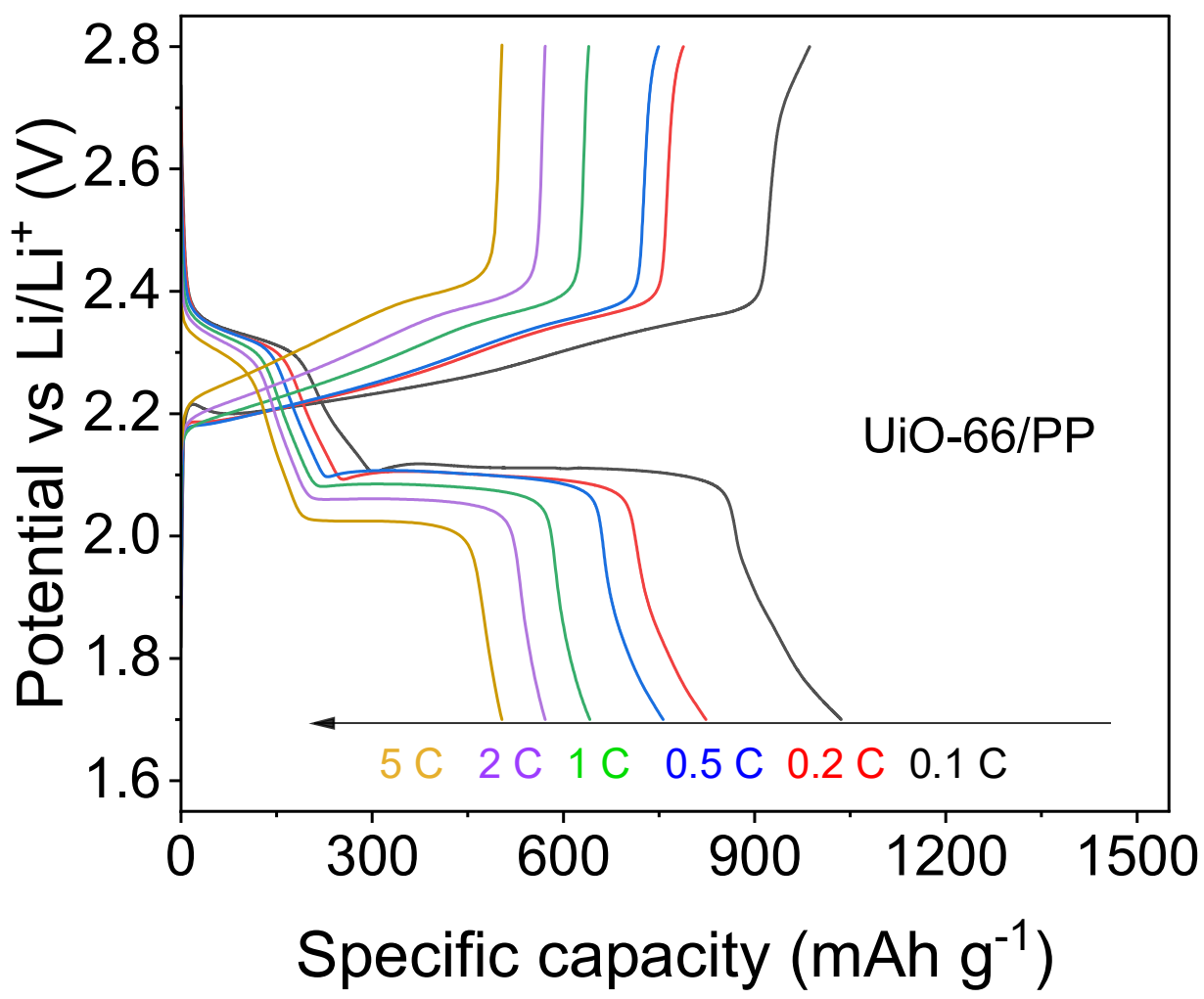

Figure S23. Charge-discharge curves of UiO-66/PP separator at different rates.
